# The effect of parental enhancing program with mobile application on parental stress and competence among Thai adolescent postpartum women: A quasi-experimental matched control design

**DOI:** 10.1371/journal.pone.0324318

**Published:** 2025-10-31

**Authors:** Sunee Kleebpan, Pornpimol Apartsakun, Pensiri Chaiyanusak, Ellen Kitson-Reynolds

**Affiliations:** 1 Department of Maternal Newborn Nursing and Midwifery, Srisavarindhira Thai Red Cross Institute of Nursing, Pathumwan, Bangkok, Thailand; 2 Obstetric ward, Queen Savang Vadhana Memorial Hospital, Sriracha, Chonburi, Thailand; 3 School of Health Sciences, University of Southampton, Highfield, Southampton, United Kingdom; National Research Centre, EGYPT

## Abstract

**Background:**

Adolescent pregnancy constitutes a critical public health issue worldwide. Young mothers face substantial physical and psychological changes as they transition to motherhood, while limited knowledge, experience, and maturity may impair parenting and increase stress. The study aimed to evaluate the effects of the parental enhancing program with mobile application on parental stress and competence among Thai adolescent postpartum women.

**Methods:**

A quasi-experimental study using a matched control design was conducted among sixty adolescent postpartum women, aged 15−19. Participants were sequentially allocated to an intervention group (n = 30) receiving a parental enhancing program with mobile application, and a control group (n = 30) receiving standard postpartum care. Adolescent postpartum women in the intervention group received two sessions from the parental enhancing program before discharge with weekly follow-up through the Line Official Account™. Data were collected using questionnaires, including the Edinburgh Postnatal Depression Scale (EPDS), Parenting Stress Index (PSI-4-SF), and Parenting Sense of Competence Scale (PSOC). Evaluations were conducted during the 6-week postpartum. ANCOVA was used to compare post-intervention mean scores of parental stress and competence between groups.

**Results:**

All enrolled participants completed the study (Intervention: 30; Control: 30). At 6-weeks postpartum, after controlling baseline EPDS scores, the results revealed that adolescent postpartum women in the intervention group demonstrated significantly lower parental stress (F(1,57) = 14.40, partial η² = 0.20, 95% CI [−14.05, −4.34], p < .001) and higher parental competence (F(1,57) = 8.79, partial η² = 0.13, 95% CI [1.90, 9.80], p < .01) compared to the control group.

**Conclusion:**

The parental enhancing program with mobile application demonstrates promise for evidence of reducing parental stress and improving parental competence in postpartum adolescent mothers. However, further studies in randomized controlled trials are warranted to confirm its effectiveness and generalizability.

**Trial registration:**

Thai clinical trials registry (TCTR20250309010).

## Introduction

Adolescent pregnancy is a major global public health concern, with significant impacts on maternal and child health outcomes. According to the United Nations’ Sustainable Development Goals (SDG) progress report, the global adolescent birth rate declined from 43.3 births per 1,000 women in 2019 to 41.3 in 2023 [1]. In Thailand, the adolescent birth rate declined from 31.3 births per 1,000 women in 2019 to 21.0 in 2022 [2]. This decline can be attributed to improved contraceptive support and increased accessibility to comprehensive sexual education and effective contraceptive methods. Despite the declining statistics of adolescent pregnancy, it remains a critical public health issue for Thailand and worldwide.

Adolescent pregnancy remains a significant health, social, and economic challenge with widespread implications for adolescent mothers’ well-being [3]. Many of these pregnancies are unplanned, with 65.6 percent of adolescent mothers reporting an absence of readiness for the demands of parenting [4]. The transition to motherhood involves substantial physical and psychological changes, often leading to role conflict and difficulties balancing developmental needs with parenting duties [5]. Peer influence and societal norms contribute to the perception of adolescent pregnancy as normal, with many teenage mothers feeling prepared and viewing their decisions positively. It has been shown that teenage mothers tend to have a more positive attitude toward pregnancy and childbirth than a negative one [6]. However, poverty, unemployment, and limited education further complicate their situation [7], contributing to increased parental stress and reduced competence [5,8].

Parental stress involves emotional responses to parenting demands, whereas parental competence reflects a mother’s positive attitude, knowledge, and skills in child-rearing. Among adolescent mothers, high stress and low competence often coexist. This imbalance not only intensifies the challenges of childcare but also undermines their confidence and ability to fulfill the maternal role. These challenges, particularly for adolescent mothers with limited life experience, underscore the critical need for effective interventions that can simultaneously mitigate stress and bolster their confidence and skills in parenting. In this context, the need for support and educational resources to reduce stress and enhance competence for adolescent postpartum women [9], nurses play a crucial role by adopting a supportive attitude and fostering open communication to address the unique needs of this population [10,11].

Evidence shows that maternal stress can be reduced by seeking guidance from family and friends, learning through clinical interactions, consulting with experienced peers, and accessing information online [12]. Therefore, effective care should focus on role adaptation through education, skills training, and counseling via online platforms. A review of the literature, along with observations from the researcher’s experience in postpartum care, reveals that adolescent mothers often receive care similar to that provided to adult mothers, using lectures and skill demonstrations. Adolescent mothers lack convenient and engaging methods to revisit parenting information, as educational materials are frequently presented in less accessible formats, such as manuals, brochures, or CDs. Furthermore, options for timely consultation are limited, presenting barriers to effective support [13–17].

Evidence from research on mobile health (mHealth) interventions has demonstrated promising outcomes in maternal childcare [18–20]. Adolescents increasingly rely on mobile applications and social media for health information, making these tools integral to modern care models [21,22]. Despite the recognized potential of mHealth interventions for maternal childcare and the high prevalence of smartphone use among adolescents, there remains a notable dearth of culturally appropriate, accessible, and engaging digital health interventions and classes specifically tailored for adolescent mothers in low- and middle-income countries (LMICs) like Thailand [23].

Traditional educational methods often fail to resonate with the digital native generation, leading to suboptimal engagement and knowledge retention. Furthermore, existing digital resources, where available, may not always address the unique psychosocial and developmental needs of adolescent mothers effectively. Recent local research highlights that Thai adolescent mothers perceive mobile applications as valuable and easily accessible sources of information [24–26], further affirming the potential of digital platforms in this context. Addressing this critical gap, our study aims to develop and evaluate a novel parental enhancing program leveraging a mobile application to provide targeted and accessible support.

To address this gap, the researchers developed a parental enhancing program with mobile application through ‘Line Official Account™ Parent Paplearn’. The platform delivers information through infographics, e-books, and videos, serving as readily accessible resources for review and offering a reliable platform for ongoing consultation. The program’s objective is to enhance parenting competence and reduce parenting stress, empowering adolescent mothers with the knowledge and confidence of motherhood.

### Research framework

This study applies Belsky’s “A Process Model of Competent Parental Functioning” [27] to elucidate the various factors influencing parental roles in childcare. This model identifies three domains, including 1) ‘Personal psychological resources of parents’ domain encompasses individual characteristics. It has been found to exert the most significant influence on parental functioning. 2) ‘Contextual sources of stress and support’, which can either facilitate or hinder effective parenting, and 3) ‘Characteristics of the Child’, which may also influence parenting dynamics but generally have a lesser impact compared to the other two domains.

The researchers utilized the parental competence framework to design the parental enhancing program with mobile application through ‘Line Official Account™ Parent Paplearn’. This program specifically targets contextual sources of stress and support, positioning nurses as vital resources to assist adolescent mothers in effectively caring for their children. In Thailand, nurses play a pivotal role in providing care through the nursing process, providing clients with appropriate education, and offering counselling. The program emphasizes the provision of knowledge and training in essential childcare skills, thereby enabling these mothers to perform their parenting roles with increased competence. Furthermore, it offers continuous support via mobile application through the ‘Line Official Account™ platform’, facilitating timely responses to questions and concerns. This ongoing support not only alleviates parenting-related stress but also enhances the mothers’ overall competence in childcare (S1 Fig).

## Materials and methods

### Study design

This study employed a non-randomized, quasi-experimental control group pre-post-test design with sequential allocation and matching to compare parental stress and competence between adolescent postpartum mothers who participated in the parental enhancing program with mobile application through ‘Line Official Account™ Parent Paplearn’ and those receiving standard postpartum care.

### Setting and samples

The study was conducted at a tertiary care hospital, Queen Savang Vadhana Memorial Hospital, Thai Red Cross Society, Thailand. The hospital has an average of 3,800 postpartum women per year, with approximately 2.5 percent being adolescent postpartum women. The study was eligible adolescent postpartum women who met the inclusion criteria; 1) postpartum women aged 15–19 who were admitted to the general obstetrics ward, 2) full-term deliveries, 3) without postpartum complications for either mother or infant, 4) intended to childcare for six weeks, and 5) demonstrated the ability to communicate via mobile using the Line application. The sample size for this study was determined using G*Power (version 3.1.9.4); the effect size was set at 0.80, following Cohen’s guidelines, a power of test equal to 0.80, and a significance level set at 0.05. The analysis indicated a sample size of 26 participants per group. To account for dropouts of 10 percent, the final sample was 30 participants per group. Consequently, the total sample size was established at 60 participants.

### Research instruments

#### Instruments for implementation.

**The parental enhancing program**, developed by the researcher, is based on Belsky’s “A Process Model of Competent Parental Functioning” [27]. The program consists of the following activities:

Session 1 (24–48 hours postpartum): Establishes rapport and strengthens the mother-infant bond. The researcher provides personalized knowledge and skills training on recognizing infant cues and responsiveness, general infant care, breastfeeding techniques, bathing procedures, and identifying abnormal symptoms in infants, lasting about 60–90 minutes. Teaching materials, such as infographics, e-books, and instructional videos, are disseminated through the ‘Line Official Account™ Parent Paplearn’ as a mobile application.

Session 2 (48–72 hours postpartum): Review the previously covered knowledge and offer additional skills training aimed at enhancing the mother’s confidence. Individualized support is provided to address her specific concerns, lasting about 60 minutes.

Session 3 (Post-discharge follow-up): Following discharge, the program includes weekly follow-up messages sent via mobile application through the ‘Line Official Account™ Parent Paplearn’ for five weeks to address any questions or concerns related to challenges faced by the mother or her infant.

**The Line Official Account™ Parent Paplearn** is a mobile application that facilitates the exchange of text messages, images, and videos on a broad scale. This platform also provides a private channel for adolescent postpartum women, enabling them to send messages for consultations or discussions regarding their parenting concerns. The platform is an online tool designed to enable users to create accounts, customize application designs, manage content, and perform system administration functions. The content available on ‘Line Official Account™ Parent Paplearn’ encompasses essential topics such as recognizing infant cues and responsiveness, general infant care, breastfeeding practices, bathing techniques, and identifying abnormal symptoms in infants. This information is delivered through engaging formats, including infographics, e-books, and videos, designed to enhance understanding and retention among adolescent mothers. The researchers developed the application interface, developed content, and primarily managed the system to address questions from adolescent postpartum women. Participants can review the knowledge as needed, without any limitation on the number of times.

#### Instruments for data collection.

**The demographic characteristics questionnaire** collects data on the mother’s age, marital status, education level, occupation, household income (THB), sufficiency of income, family structure, desire for children, plan for future education, childcare support, experience in childcare, gravida, type of delivery, and the infant’s APGAR score.

**The postpartum problem and advice record** was developed by the researcher to systematically document follow-up interactions via mobile application through the ‘Line Official Account™ Parent Paplearn’ after hospital discharge, recording the date, problem, and advice provided.

**The Edinburgh Postnatal Depression Scale (EPDS)** was translated into Thai by Vacharaporn et al. [28] This screening tool consists of 10 items. For example, I have been able to laugh and see the funny side of things, I have looked forward with enjoyment to things, etc. The total scores range from 0 to 30 points. A cut-off score of 11 or higher indicates potential postpartum depression. The EPDS serves as an important instrument in assessing the mental health needs of this population, facilitating timely interventions to promote overall well-being.

**The Parenting Stress Index Fourth Edition Short Form (PSI-4-SF)** utilized in this study employed the Thai version developed by Srikosa et al. [29] Originally 36 items, it was reduced to 15 to align with the context of the research, specifically focusing on infants aged six weeks postpartum. It measures three domains, including 1) Parental distress, 2) Parent-child dysfunctional interaction, and 3) Difficult child, utilizing a five-point Likert scale. Scores range from 15 to 75 points, where lower scores indicate lower levels of parenting stress, while higher scores reflect greater parenting stress. The instrument demonstrated strong validity (CVI = 0.94) and reliability (Cronbach’s alpha = 0.78).

**The Parenting Sense of Competence Scale (PSOC)** was translated into Thai by Kleebpan et al. [30]. This scale consists of 16 items measuring parenting efficacy and satisfaction. For example, the problems of taking care of a child are easy to solve once you know how your actions affect your child, an understanding you have acquired, etc. Scores ranged from 16 to 96 points. In this context, lower scores indicate lower levels of parenting competence, while higher scores reflect greater competence in parenting. The instrument demonstrated strong validity (CVI = 0.81) and reliability (Cronbach’s alpha = 0.72).

### Ethical considerations

This study received ethical approval from the Ethics Committee of Queen Savang Vadhana Memorial Hospital, Thai Red Cross Society (COE No. 001/2566) on February 6, 2023. The study was retrospectively registered on the Thai clinical trials registry (TCTR20250309010; Date of registration: 09/03/2025), as due to the COVID-19 pandemic, operations at the institution were suspended. Additionally, in November 2023, the institution temporarily stopped its operation. These circumstances prevented the authors from completing prospective registration within the required timeframe. Retrospective registration was therefore undertaken to ensure transparency, reduce potential reporting bias, and comply with international guidelines of the ICMJE and WHO. The authors confirm that all ongoing and related trials for this intervention are properly registered and adhere to the TREND checklist (S1 Checklist) as specified in the study protocol (S1 and S2 Files), with no alterations to the primary objectives or methods following study initiation.

Participants were informed about the study, assured of confidentiality, and provided written consent, with parental or spousal consent required for those under 18. Participation was voluntary, with the right to withdraw at any time without any consequence. Participants scoring 11 or higher on the EPDS or indicating suicidal thoughts were not excluded. Instead, the researcher provided initial support by actively listening to their concerns and referred them to a nurse who facilitated further consultation with a psychiatrist, ensuring that they received appropriate emotional and mental health care.

### Data collection procedure

All participants were recruited between 14 February 2023 and 21 June 2024, with each participating in the study for a duration of six weeks. Data collection for the final participant was completed on 2 August 2024. The study was conducted sequentially, beginning with the control group, which was recruited from 14 February to 11 August 2023. The final follow-up for this group was completed on 22 September 2023. Subsequently, the intervention group was recruited from 26 August 2023 to 21 June 2024, with the final follow-up completed on 2 August 2024. Participants were matched based on gravidity, desire for children, marital status, experience in childcare, and childcare support. Matched participants were then assigned to the intervention group accordingly.

A sequential design was employed in this study, with participants recruited and assigned to groups in a time-ordered manner. This approach aimed to reduce the influence of external confounding variables that may vary over time, such as staffing changes or institutional policies. By separating group recruitment periods, the investigators sought to minimize overlap and potential contamination between groups. However, it is acknowledged that this design does not eliminate all sources of bias, and the lack of randomization remains a limitation.

The researcher collaborated with nurses to identify eligible participants who met the inclusion criteria and obtained permission to meet them after 24 hours postpartum. During these meetings, the researcher explained the study objectives, procedures, and participants’ rights. Informed consent forms were then obtained from the participants. Before the intervention, the researcher administered several assessment tools to collect baseline data, including the demographic characteristics questionnaire, the Edinburgh Postnatal Depression Scale [28], the Parenting Stress Index Fourth Edition Short Form [29], and the Parenting Sense of Competence Scale [30]. After that, all participants were scheduled for intervention, while the control group had standard postpartum care. This process will continue until the required sample size is reached, with 30 participants in each group.

At one week postpartum, participants in both the intervention and control groups completed the Edinburgh Postnatal Depression Scale [28] to screen for postpartum depression, ensuring that appropriate emotional and psychological support could be provided based on the findings. In cases where no baseline differences were detected between groups, the researchers planned to adjust for variable variance using appropriate statistical methods.

**Fig 1:**
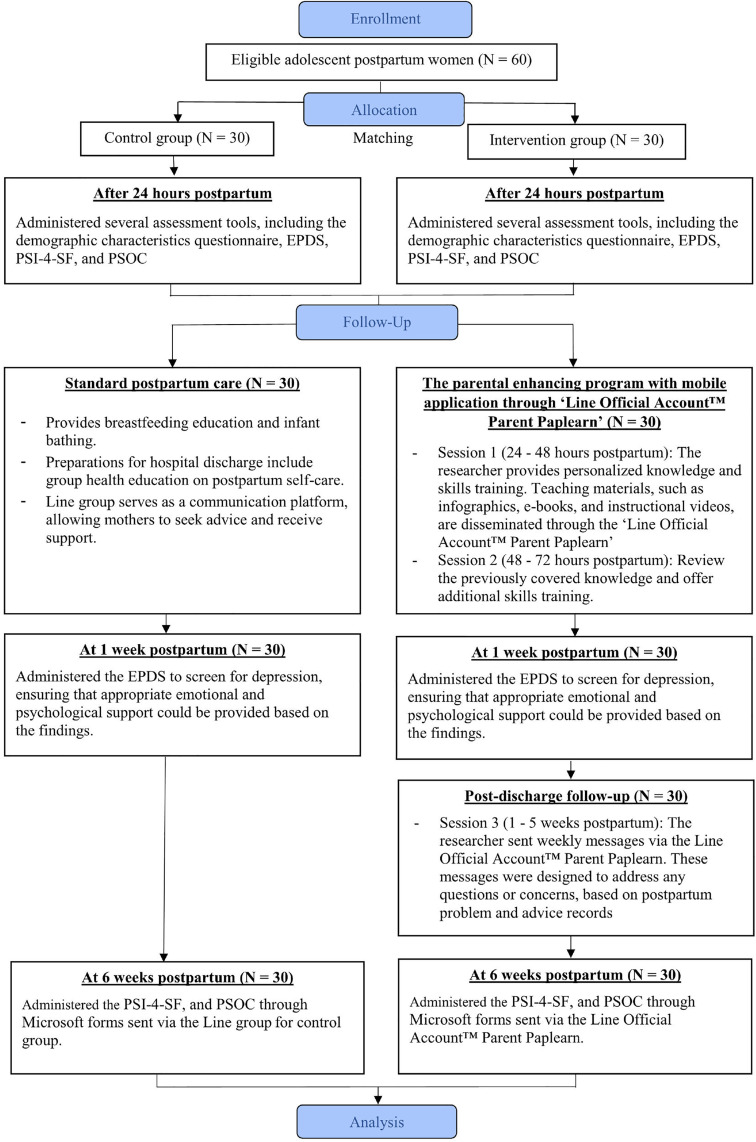
Flow diagram of study.

Then, the researcher sent weekly messages via a mobile application through the Line Official Account™ Parent Paplearn for a duration of five weeks. These messages were designed to address any questions or concerns regarding the challenges participants faced in child-rearing and parenting responsibilities after discharge, based on postpartum problem and advice records. At the conclusion of the study, six weeks postpartum, participants in both groups were administered the Parenting Stress Index Fourth Edition Short Form [29] and the Parenting Sense of Competence Scale [30]. Details of the study as shown in Fig 1.

### Statistical analysis

IBM SPSS Statistics Version 28 (License code: 5f5119869c4164e0bda0) was used for data analysis. Descriptive statistics were used to characterize the participant demographics and obstetric characteristics. The comparison of differences between the characteristics of the intervention group and the control group was conducted using an independent t-test and a chi-square test based on the type of variables and assumptions of statistical significance. Prior to data analysis, the normality of data distribution and homogeneity of variance were assessed using the Kolmogorov-Smirnov test and Levene’s test, respectively. The paired t-test was used to compare pre-post intervention mean scores within each group. To compare mean scores of parental stress and competence between groups, ANCOVA will be conducted, controlling for baseline differences in EPDS score at 1 week postpartum as a covariate. Statistical significance was set at p < .05. Additionally, effect sizes, partial η², and 95% confidence intervals will be reported to indicate the practical significance of the findings.

## Results

In the study 30 adolescent postpartum women in each group. In the intervention group, one participant had an APGAR score of 6 at one minute but a normal score at five minutes without complications; thus, all participants met the inclusion criteria. Tests of homogeneity revealed no significant differences between the intervention and control groups in all demographics and obstetric characteristics, except for the significant difference in EPDS at 1 week postpartum (2.63 ± 4.21 vs 6.57 ± 5.82, p < .05). The baseline imbalance in EPDS scores at 1 week was carefully addressed in the ANCOVA to control for baseline differences. The details are shown in Table 1.

**Table 1:** Participant demographics and obstetric characteristics (N = 60).

Variables	Intervention group (n = 30)		Control group (n = 30)		p-value
	n	%	n	%	
**Age (years)**	(Mean =** **18.27, SD = 0.87)		(Mean =** **18.17, SD = 1.17)		0.71^a^
16	1	3.3	5	16.7	
17	5	16.7	3	10.0	
18	9	30.0	4	13.3	
19	15	50.0	18	60.0	
**Marital status**					0.58^b^
Married	26	86.7	28	93.4	
Separated	3	10.0	1	3.3	
Divorced	1	3.3	1	3.3	
**Education level**					0.36^b^
Elementary school	2	6.7	3	10.0	
Secondary school	27	90.0	27	90.0	
Bachelor’s degrees (In progress)	1	3.3	0	0.0	
**Occupation**					0.38^b^
Unemployed	15	50.0	12	40.0	
Employee	13	43.4	12	40.0	
Merchant	1	3.3	4	13.3	
Housewife	1	3.3	2	6.7	
**Household income (THB)**	(Mean =** **17,833.33, SD = 9,172.99)		(Mean =** **16,289.33, SD = 8,673.57)		0.51^a^
< 10,000	4	13.3	6	20.0	
10,000 - 20,000	20	66.7	17	56.6	
20,001 - 30,000	4	13.3	5	16.7	
> 30,000	2	6.7	2	6.7	
**Sufficiency of income**					0.50^b^
Sufficient	22	73.4	23	76.7	
Insufficient	8	26.6	7	23.3	
**Family structure**					1.00^b^
Nuclear	14	46.7	14	46.7	
Extended	15	50.0	15	50.0	
Single mother	1	3.3	1	3.3	
**Desire for children**					0.37^b^
Yes	24	80.0	26	86.7	
No	6	20.0	4	13.3	
**Plan for further education**					0.29^b^
Immediately	6	20.0	2	6.7	
Later	8	26.6	14	46.7	
Not continuing	7	23.3	6	20.0	
Uncertain	9	30.0	8	26.6	
**Childcare support**					0.34^b^
No assistance	2	6.7	4	13.3	
With assistance	28	93.4	26	86.7	
**Experience in childcare**					0.13^b^
No experience	24	80.0	19	63.3	
Experienced	6	20.0	11	36.7	
**Gravida**					0.50^b^
1	26	86.7	27	90.0	
> 1	4	13.3	3	10.0	
**Type of delivery**					0.30^b^
Vaginal delivery	15	50.0	23	76.7	
Cesarean section	15	50.0	7	23.3	
**Birth weight (grams)**	(Mean = 2,940.00, SD = 418.49)		(Mean = 3,020.33, SD = 309.11)		0.40^a^
< 2,500	5	16.7	2	6.7	
≥ 2,500	25	83.3	28	93.4	
**APGAR score at 1 minute**	(Mean = 8.47, SD = 0.68)		(Mean = 8.70, SD = 0.53)		0.15^a^
0-3	0	0.0	0	0.0	
4-6	1	3.3	0	0.0	
7-10	29	96.7	30	100.0	
**APGAR score at 5 minutes**	(Mean = 9.07, SD = 0.25)		(Mean = 9.07, SD = 0.25)		1.00^a^
0-3	0	0.0	0	0.0	
4-6	0	0.0	0	0.0	
7-10	30	100.0	30	100.0	
**EPDS score at 1 day postpartum**	(Mean = 8.20, SD = 5.51)		(Mean = 7.33, SD = 4.40)		0.50^a^
< 11	20	66.7	23	76.7	
≥ 11	10	33.3	7	23.3	
**EPDS score at 1 week postpartum**	(Mean = 2.63, SD = 4.21)		(Mean = 6.57, SD = 5.82)		**0.00** ^a*^
< 11	29	96.7	24	80.0	
≥ 11	1	3.3	6	20.0	

SD, standard deviation; EPDS, The Edinburgh Postnatal Depression Scale. A score of <11 indicates no clinical concern for postpartum depression, while a score of ≥ 11 suggests possible postpartum depression.

^a^Independent t-test,

^b^Chi-square test,

*statistically significant is at p < .05

The paired t-test revealed a statistically significant difference in the intervention group after the parental enhancing program with mobile application through ‘Line Official Account™ Parent Paplearn’. Parental stress significantly decreased (p < .01), while parental competence increased (p < .001). In contrast, the control group showed no statistically significant differences in either parental stress (p > .05) or competence (p > .05), as detailed in Table 2.

**Table 2:** Mean scores of parental stress and parental competence before and after the intervention within each group (N = 60).

Variables	Before intervention		After intervention		Mean difference	t-test	p-value
	Mean	SD	Mean	SD			
**Parental stress**							
Intervention group (n = 30)	37.63	7.10	32.47	4.88	5.17	3.46	0.002*
Control group (n = 30)	39.27	7.74	42.63	11.67	−3.37	−1.67	0.107
**Parental competency**							
Intervention group (n = 30)	62.77	8.45	70.87	6.49	−8.10	−4.55	0.000*
Control group (n = 30)	63.90	6.74	62.57	8.79	1.33	0.87	0.393

SD, standard deviation.

*statistically significant is at p < .01

At 6-weeks postpartum, after controlling for baseline EPDS scores, the intervention group demonstrated significantly lower adjusted mean scores of parental stress (Adjusted Mean = 32.95, SE = 1.66) compared to the control group (Adjusted Mean = 42.15, SE = 1.66), F(1, 57) = 14.40, p < .001, with a large effect size (partial η² = 0.20, 95% CI for adjusted mean difference = [−14.05, −4.34). Similarly, adjusted mean scores of parental competence were significantly higher in the intervention group (Adjusted Mean = 69.64, SE = 1.35) than in the control group (Adjusted Mean = 63.79, SE = 1.35), F(1, 59) = 8.79, p < .01, with a moderate-large effect size (partial η² = 0.13, 95% CI for adjusted mean difference = 1.90, 9.80). These detailed results are presented in Table 3.

**Table 3:** Adjusted parental stress and parental competence scores between intervention groups and control groups after intervention (6 weeks postpartum), when controlling for baseline EPDS score at 1 week postpartum (N = 60).

Variables	Adjusted Mean (SE)	Mean Difference (95% CI)	F (df₁, df₂)	p-value**	partial η²
**Parental stress**					
Intervention group (n = 30)	32.95 (1.66)	−9.20 (−14.05,-4.34)	14.40 (1,57)	0.000*	0.20
Control group (n = 30)	42.15 (1.66)				
**Parental competency**					
Intervention group (n = 30)	69.64 (1.35)	5.85 (1.90,9.80)	8.79 (1,57)	0.004*	0.13
Control group (n = 30)	63.79 (1.35)				

SE, standard error of the mean; CI, confidence interval; df₁, numerator degrees of freedom; df₂, denominator degrees of freedom; Partial η², partial eta squared (effect size).

*statistically significant is at p < .01

**adjusted by ANCOVA with multiple comparisons using Bonferroni test.

## Discussion

The findings of this quasi-experimental study indicated that participants in the intervention group reported lower parental stress and higher parental competence compared with the control group. While these results are promising and consistent with the research hypothesis, they should be interpreted cautiously as preliminary evidence of the program’s potential benefits, rather than conclusive proof of effectiveness. Future randomized controlled trials with larger and more diverse samples are needed to validate these findings.

The parental enhancing program with mobile application through ‘Line Official Account™ Parent Paplearn’, was developed based on Belsky’s “A Process Model of Competent Parental Functioning” [27]. The program was designed to address contextual sources of stress and support, with nurses providing adolescent mothers with childcare knowledge, skill training, and ongoing support through the Line platform. The observed improvements are consistent with previous evidence. For instance, the study by Léniz-Maturana et al. [31] demonstrated that maternal role confidence is associated with reduced stress levels. Similarly, Flaherty et al. [32] found that parental competence and perceived social support play a critical role in moderating stress levels. These converging findings suggest that structured support may assist adolescent mothers during their transition to parenthood.

The findings are consistent with previous studies on parental programs that demonstrated improvements in maternal outcomes. For example, Saleh et al. [17] reported that parenting coaching interventions for teenage mothers reduced stress and enhanced knowledge, attitudes, behaviors, and self-efficacy. Similarly, Kordi et al. [14] emphasized the importance of maternal role training in fostering role fulfillment and satisfaction, while Moudi et al. [15] found that even brief face-to-face parenting skills training significantly improved perceived self-efficacy and mother–infant bonding. In addition, Polgaya et al. [16] highlighted the benefits of continuous support, including repeated skills training, regular assessments, and follow-up consultations, can strengthen maternal attitudes and performance. While the present program incorporates ongoing support through the Line platform, the extent to which it replicates or exceeds the benefits of these interventions requires further investigation.

This study also contributes to the growing literature on mobile technology–based interventions for parenting support. Prior research has demonstrated the potential of such approaches. Shorey et al. [19] demonstrated that telephone-based interventions and mobile health app follow-up enhance parenting self-efficacy, bonding, perceived social support, and satisfaction, while also reducing postnatal depression. Chua et al. [18] indicated that mobile applications improved parenting self-efficacy, anxiety, depression, social support, and bonding. Additionally, Nuampa et al. [33] indicated that adolescent mothers perceive mobile applications as accessible sources, with nurses acknowledging that these applications can reduce their workload, improve preparedness, and enhance the standard of support provided. The current findings suggest that integrating mobile technology may improve accessibility and engagement for adolescent mothers, though further studies are needed to confirm this modernizing health promotion.

### Study limitations

Despite the encouraging findings, this study has several important limitations that must be considered. First, the study employed a non-randomized, quasi-experimental design with sequential allocation and matching, while intended to minimize confounding, restricts the ability to establish causality compared with randomized controlled trials. Second, the retrospective registration might introduce concerns regarding potential reporting bias and the prospective nature of the study design, thereby potentially affecting the overall credibility of the findings. Third, a significant baseline difference in EPDS scores. Although statistical adjustments were applied, this imbalance may still influence the interpretation of between-group comparisons. Fourth, the reliance on self-report instruments for outcome measures raises the possibility of response bias, which may affect the accuracy of the findings. Finally, practical implementation of the program requires time and involvement from nurses, as well as participants’ access to smartphones and familiarity with the Line application, which may limit generalizability in settings with fewer resources.

## Conclusion

The parental enhancing program with a mobile application demonstrates promise in supporting postpartum adolescent mothers by reducing parental stress and improving parental competence. However, as this study used a quasi-experimental design, future studies employing randomized controlled designs are warranted to confirm these findings and strengthen the evidence base for practice.

## Supporting information

S1 Fig Research framework. (TIF)

S1 Checklist TREND checklist. (DOCX)

S1 File Protocol. Copy of the study protocol (Original version).
(PDF)

S2 File Protocol. Study protocol (English translation version).
(PDF)
