# Supplementary figures and images for: The effect of parental enhancing program with mobile application on parental stress and competence among Thai adolescent postpartum women: A quasi-experimental matched control design
Source: PLoS One. 2025 Oct 31;20(10):e0324318. doi: 10.1371/journal.pone.0324318 (PMC12578233; doi:10.1371/journal.pone.0324318)

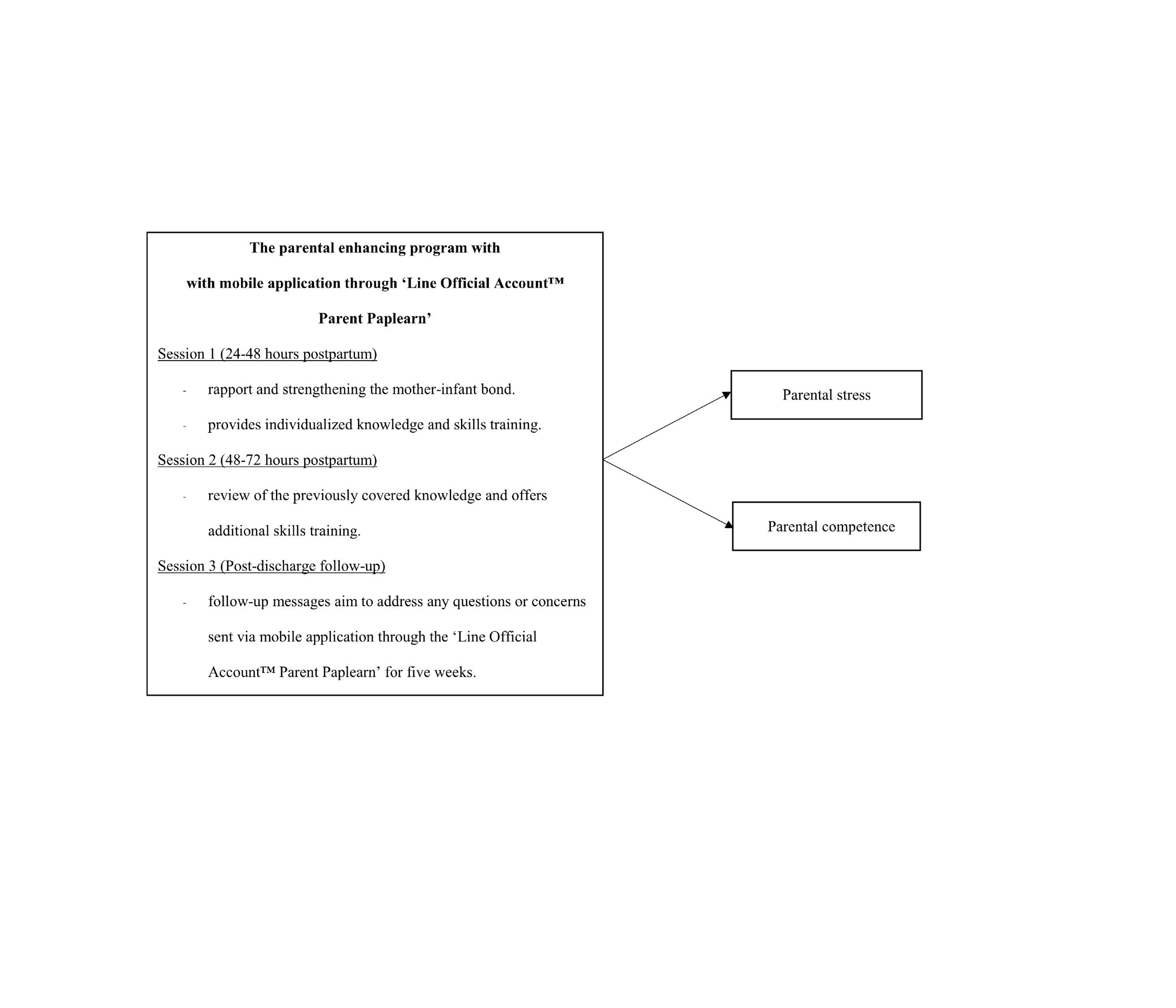

Supplement: S1 Fig — (TIF) [file pone.0324318.s001.tif]
