# Supplementary material for: The effect of parental enhancing program with mobile application on parental stress and competence among Thai adolescent postpartum women: A quasi-experimental matched control design
Source: PLoS One. 2025 Oct 31;20(10):e0324318. doi: 10.1371/journal.pone.0324318 (PMC12578233; doi:10.1371/journal.pone.0324318)
Supplement: S1 File — Copy of the study protocol (Original version). (PDF) [file pone.0324318.s003.pdf]

โครงร่างการวิจัย (Research Protocol)

1. ชื่อเรื่องการวิจัย (Research title)

(ภาษาไทย) ผลของโปรแกรมส่งเสริมการเลี้ยงดูบุตรร่วมกับการใช้ Line Official Account "Parent พาเพลิน" ต่อความเครียดในการเลี้ยงดูบุตร และสมรรถนะในการเลี้ยงดูบุตรของมารดาวัยรุ่นหลังคลอด

(ภาษาอังกฤษ) The Effect of Parental Enhancing Program with Line Official Account "Parent Paplearn" on Parental Stress and Parental Competence Among Adolescent Postpartum Mothers

2. ชื่อคณะผู้วิจัย (Investigators)

2.1 ผู้วิจัยหลัก (Principal investigator)

ชื่อ-สกุล อาจารย์สุนีย์ กลีบปาน ตำแหน่ง อาจารย์ สาขาวิชาการพยาบาลมารดา ทารกและการผดุงครรภ์ สถาบันการพยาบาลศรีสวรินทิรา สภากาชาดไทย เบอร์โทรศัพท์ 092-461-4926

2.2 ผู้ร่วมวิจัย (Co-investigators)

ชื่อ-สกุล ผศ. ดร. พรพิมล อาภาสสกุล ตำแหน่ง ผู้ช่วยศาสตราจารย์ สาขาวิชาการพยาบาลมารดา ทารกและการผดุงครรภ์ สถาบันการพยาบาลศรีสวรินทิรา สภากาชาดไทย เบอร์โทรศัพท์ 096-883-6024

2.3 ผู้ร่วมวิจัย (Co-investigators)

ชื่อ-สกุล นางเพ็ญศิริ ไชยานุศักดิ์ ตำแหน่ง พยาบาลปฏิบัติการ 7 หอผู้ป่วยสูติกรรมสามัญ 18A โรงพยาบาลสมเด็จพระบรมราชเทวี ณ ศรีราชา สภากาชาดไทย เบอร์โทรศัพท์ 038-320-200

2.4 ที่ปรึกษาโครงการ

ชื่อ-สกุล รศ. ดร. นิตยา สินสุกใส ตำแหน่ง รองศาสตราจารย์ ภาควิชาการพยาบาลสูติศาสตร์-นรีเวชวิทยา คณะพยาบาลศาสตร์ มหาวิทยาลัยมหิดล เบอร์โทรศัพท์ 02-4197477 ต่อ 1804

3. ที่มาและความสำคัญของปัญหา (Background and rationale)

การตั้งครรภ์วัยรุ่นเป็นปัญหาทางสาธารณสุขที่ทั่วโลกรวมทั้งประเทศไทยต่างให้ความสำคัญ จากรายงานความคืบหน้าเป้าหมายการพัฒนาที่ยั่งยืนขององค์การสหประชาชาติ พบว่าการคลอดในหญิงตั้งครรภ์วัยรุ่นมีแนวโน้มที่ลดลง โดยอัตราการคลอดในหญิงตั้งครรภ์วัยรุ่นทั่วโลกต่อการเกิดมีชีพ 1,000 ราย ปี ค.ศ. 2015 พบ 45 ราย และในปี ค.ศ.2019 พบ 44 ราย (United Nations, 2019) ในสหรัฐอเมริกาพบอัตราการคลอดในหญิงตั้งครรภ์วัยรุ่นอายุ 15-19 ปีต่อประชากรหญิงอายุ 15-19 ปี 1,000 ราย โดยในปี ค.ศ. 2018 = 17.4 และปี ค.ศ. 2019 = 16.7 (Martin, Hamilton & Osterman, 2021) ในส่วนของประเทศไทยนั้นจากสถิติในช่วง พ.ศ. 2558-2562 พบว่าอัตราการคลอดในหญิงตั้งครรภ์วัยรุ่นอายุ 15-19 ปี นั้นมีอัตราการคลอด 44.8, 42.5, 39.6, 35.0 และ 31.3 ของประชากรหญิง 15-19 ปี 1,000 รายตามลำดับ (สำนักอนามัยการเจริญพันธุ์, 2563) จากสถิติข้างต้นนั้นถึงแม้จะมีแนวโน้มลดลง แต่จะเห็นได้ว่าอัตราการคลอดในหญิงตั้งครรภ์วัยรุ่นนั้นยังไม่บรรลุเป้าหมายการพัฒนาที่ยั่งยืน (Sustainable Development Goals, SDGs) ในเป้าหมายที่ 3 กำหนดให้ลดอัตราคลอดในวัยรุ่นอายุ 10-19 ปี ภายในปี 2573 และเป้าหมายของยุทธศาสตร์การป้องกันและแก้ไขปัญหาการตั้งครรภ์ในวัยรุ่น ระดับชาติ พ.ศ. 2560-2569 ที่มีเป้าหมายภายในปี 2569 ให้ลดอัตราคลอดในวัยรุ่นอายุ 15-19 ปี ไม่เกิน 25 ของประชากรหญิง 15-19 ปี 1,000 ราย (สำนักอนามัยการเจริญพันธุ์, 2560)

จากสถานการณ์การตั้งครรภ์วัยรุ่นในปัจจุบันที่ยังมีอัตราที่สูง อีกทั้งยังไม่บรรลุตามเป้าหมายของระดับโลกและประเทศชาติ และเป็นปัญหาที่ส่งผลกระทบทั้งทางด้านสุขภาพของมารดา ทารก รวมถึงสังคมและเศรษฐกิจของประเทศในภาพรวม มารดาวัยรุ่นต้องเผชิญกับการเปลี่ยนแปลงต่าง ๆ หลายประการทั้งทางด้านร่างกาย ด้านจิตใจ และสิ่งสำคัญคือการเปลี่ยนผ่านสู่บทบาทใหม่ นั่นคือบทบาทการเป็นมารดา มารดาวัยรุ่นต้องรับผิดชอบมากขึ้น มีความกลัวและความวิตกกังวลเกี่ยวกับการเลี้ยงดูบุตร (Mangeli, Rayyani, Cheraghi, & Tirgari, 2017) นอกจากนี้

INSTITUTION REVIEW BOARD
Queen Savang Vadhana Memorial Hospital
IRB No. ......01....... /.....2566..........
Date of Approval ....- 6 ก.พ. 2566....

การขาดความรู้ และประสบการณ์ในการเป็นมารดา วุฒิภาวะทางด้านร่างกาย จิตใจ อารมณ์ สังคมที่ยังไม่พร้อม อาจทำให้มารดารู้สึกขาดความมั่นใจในการเลี้ยงดูบุตร และมีความเครียดในการเลี้ยงดูบุตร โดยเฉพาะอย่างยิ่งในช่วงระยะแรกหลังคลอด (Ngai & Chan, 2012) ซึ่งจะส่งผลให้สมรรถนะในการเลี้ยงดูบุตรไม่มีประสิทธิภาพ

ความเครียดในการเลี้ยงดูบุตร (parental stress) เป็นปฏิกิริยาการตอบสนองทางอารมณ์ ซึ่งเกิดจากการรับรู้ หรือการแปลความหมายจากสถานการณ์ต่าง ๆ ในการเลี้ยงดูบุตรที่เข้ามากระทบ การเลี้ยงดูบุตรเป็นบทบาทใหม่ของมารดาวัยรุ่นที่ต้องเรียนรู้ โดยเฉพาะอย่างยิ่งในระยะหลังคลอด เป็นระยะแห่งการเรียนรู้วิธีในการเลี้ยงดูบุตร การสร้างสัมพันธภาพกับบุตร รวมไปถึงการเรียนรู้พฤติกรรมของบุตร หากมารดาวัยรุ่นไม่สามารถทำหน้าที่ในการเลี้ยงดูบุตรได้ตามความคาดหวังของตนเองและบุคคลรอบข้าง อาจเกิดความรู้สึกไม่สบายใจ วิตกกังวล ซึ่งจะส่งผลต่อการปรับตัวในการทำหน้าที่เลี้ยงดูบุตรของมารดาวัยรุ่น การที่จะสามารถลดความเครียดได้โดยการขอคำแนะนำจากผู้ใกล้ชิด, การสอนในหอผู้ป่วย, สอบถามเพื่อนที่มีประสบการณ์ และการอ่านข้อมูลทางสื่อออนไลน์ (สุริยา ยอดทอง, รุ่งฤดี อุสาหะ และอาภรณ์ ภู่พัทธยากร, 2563)

สมรรถนะในการเลี้ยงดูบุตร (parental competence) เป็นสิ่งที่บ่งบอกถึงการที่มารดามีทัศนคติที่ดี มีความรู้เกี่ยวกับการดูแลบุตร มีทักษะและความสามารถที่เกี่ยวกับการเลี้ยงดูบุตรที่ดี ซึ่งมารดาจะสามารถจัดการและปฏิบัติงานเกี่ยวกับการดูแลบุตรได้อย่างมีประสิทธิภาพจนเกิดความพึงพอใจในการแสดงบทบาทของตนเอง แต่จะเห็นได้ว่า มารดาวัยรุ่นนั้นจะต้องมีการปรับตัวที่ท้าทายอย่างมากทั้งในด้านความรับผิดชอบที่มากขึ้น ต้องเผชิญกับการเปลี่ยนแปลงหรือปัญหาทางด้านร่างกาย เกิดความเครียดและความวิตกกังวล การแสดงบทบาทมารดาที่ไม่มีประสิทธิภาพ และการขัดแย้งในบทบาท (Mangeli et al., 2017) ซึ่งจะส่งผลลบต่อสมรรถนะในการเลี้ยงดูบุตร โดยเฉพาะอย่างยิ่งมารดาวัยรุ่น ดังนั้นมารดาวัยรุ่นจึงเป็นกลุ่มที่สำคัญในการส่งเสริมให้มีความสามารถในการเลี้ยงดูบุตร

ในการดูแลและส่งเสริมภาวะสุขภาพของมารดาวัยรุ่น พยาบาลต้องมีทัศนคติที่ดีกับมารดาในกลุ่มนี้ และเปิดโอกาสในการสื่อสาร เพื่อที่จะสามารถเข้าใจความต้องการของมารดาวัยรุ่นได้ จะสามารถลดความวิตกกังวลเกี่ยวกับการดูแลบุตร และส่งเสริมการปรับตัวในการเป็นมารดาได้ดีขึ้น (Erfina, Widyawati, McKenna, Reisenhofer & Ismail, 2019; ศิริรัตน์ อินทรเกษม และคณะ, 2562) เน้นการส่งเสริมการปรับบทบาทของมารดา โดยการให้ความรู้ ฝึกทักษะในการเลี้ยงดูบุตรและการเลี้ยงดูบุตรด้วยนมมารดา การส่งเสริมสัมพันธภาพมารดา – ทารก เพื่อให้มารดาวัยรุ่นเกิดความมั่นใจ และในปัจจุบันเทคโนโลยีเข้ามามีบทบาทอย่างมากกับการดำเนินชีวิตประจำวันของบุคคล โดยเฉพาะอย่างยิ่งวัยรุ่นจะแสวงหาข้อมูลต่าง ๆ จากอินเตอร์เน็ตเป็นส่วนใหญ่ การเพิ่มช่องทางในการติดต่อสื่อสารผ่านสื่อโซเชียลมีเดียต่าง ๆ จะเป็นสิ่งสำคัญสำหรับรูปแบบการดูแลในปัจจุบัน (ขนิษฐา เมฆกมล และอารีรัตน์ วิเชียรประภา, 2561) แต่รูปแบบการดูแลมารดาระยะหลังคลอดในปัจจุบัน โรงพยาบาลมีนโยบายจำหน่ายผู้รับบริการเร็วขึ้น โดยส่วนใหญ่ระยะเวลาในการอยู่โรงพยาบาลจะประมาณ 2 – 3 วัน ซึ่งส่งผลให้มารดาหลังคลอดไม่สามารถเรียนรู้และฝึกทักษะในการเลี้ยงดูบุตรได้อย่างครอบคลุมและต่อเนื่อง นอกจากนี้ยังพบว่าโรงพยาบาลส่วนใหญ่ขาดการดูแลหรือช่องทางในการติดต่อสื่อสารภายหลังการจำหน่าย จะมีเพียงคู่มือในการเลี้ยงดูบุตรเพื่อให้มารดาหลังคลอดทบทวนเท่านั้น ซึ่งอาจสร้างความวิตกกังวลเมื่อมารดาต้องกลับไปเลี้ยงดูบุตรด้วยตนเองที่บ้าน และส่งผลต่อประสิทธิภาพในการเลี้ยงดูบุตร

จากการทบทวนวรรณกรรมข้างต้นประกอบกับประสบการณ์ในการดูแลมารดาในระยะหลังคลอดของผู้วิจัย พบว่ารูปแบบในการดูแลมารดาวัยรุ่นไม่แตกต่างกับมารดาในวัยผู้ใหญ่ นั่นคือการบรรยายให้ความรู้และการสาธิต ฝึกทักษะ แต่พบว่ายังขาดช่องทางในการให้มารดาวัยรุ่นได้มีการทบทวนเนื้อหาเกี่ยวกับการเลี้ยงดูบุตร ส่วนใหญ่จะเป็นการให้คู่มือ แผ่นพับ หรือ CD ซึ่งอาจทำให้มารดาวัยรุ่นไม่เกิดความสนใจ และยังขาดช่องทางในการปรึกษาที่สะดวก ด้วยเหตุนี้ผู้วิจัยจึงต้องการพัฒนารูปแบบการดูแลโดยใช้เทคโนโลยีเข้ามามีส่วนร่วมในการให้การดูแลมารดาวัยรุ่น

ปัจจุบันแอพลิเคชั่นไลน์ ถือเป็นช่องทางในการติดต่อสื่อสารที่เป็นที่นิยม จากการสำรวจพฤติกรรมการใช้แอพลิเคชั่นไลน์ของผู้ใช้ในประเทศไทย ปี 2563 พบว่า มีผู้ใช้แอพลิเคชั่นไลน์จำนวน 47 ล้านคน โดยในกลุ่ม Gen Z ใช้อินเตอร์เน็ตเฉลี่ยวันละ 12 ชั่วโมง 8 นาที (กระทรวงดิจิทัลเพื่อเศรษฐกิจและสังคม, 2563) ดังนั้นแอพลิเคชั่นไลน์จึงเป็นช่องทางที่น่าสนใจและเข้าถึงกลุ่มวัยรุ่นได้ดี โดย line official account เป็นบัญชีที่สร้างขึ้นเพื่อการส่งข้อมูลข่าวสารต่าง ๆ ในวงกว้าง สามารถที่จะเผยแพร่ข้อความ รูปภาพ วิดีโอ หรือการประชาสัมพันธ์ให้กับผู้ติดตามได้

INSTITUTION REVIEW BOARD
Queen Savang Vadhana Memorial Hospital
IRB No. ......01......... /..........2566..............
Date of Approval .... - 6 ก.พ. 2566

สะดวก นอกจากนี้ผู้ที่ติดตามสามารถส่งข้อความกลับมาเพื่อพูดคุยหรือปรึกษาได้ โดยที่บุคคลอื่น ๆ ใน line official account จะไม่เห็นข้อความดังกล่าว ซึ่งเป็นช่องทางในการติดต่อสื่อสารที่มีความเป็นส่วนตัว และไม่มีค่าใช้จ่ายเพิ่มเติมจากแพ็คเกจอินเตอร์เน็ต ดังนั้นการใช้ line official account มาเป็นสื่อกลางในการเผยแพร่ความรู้ จะเป็นประโยชน์ในการติดต่อสื่อสาร และเป็นช่องทางในการปรึกษาปัญหาของมารดาวัยรุ่นกับบุคลากรทางสุขภาพได้อย่างสะดวกและรวดเร็วยิ่งขึ้น

คณะผู้วิจัยจึงพัฒนาโปรแกรมการเลี้ยงดูบุตร โดยให้ความรู้และฝึกทักษะเกี่ยวกับการเลี้ยงดูบุตร ผ่านการให้ความรู้รายบุคคล ร่วมกับการใช้ line official account "Parent พาเพลิน" ซึ่งจะมีเนื้อหาเกี่ยวกับสื่อสัญญาณทารกและการตอบสนองต่อสื่อสัญญาณทารก การดูแลบุตร การเลี้ยงบุตรด้วยนมมารดา การอาบน้ำบุตร และการสังเกตอาการผิดปกติของทารก ในรูปแบบของ infographics, e-book, วิดีทัศน์ เป็นต้น มาเป็นสื่อกลางในการเผยแพร่ความรู้ เป็นประโยชน์ในการทบทวน และเป็นช่องทางในการปรึกษาปัญหาของมารดาวัยรุ่น เพื่อส่งเสริมให้มารดาวัยรุ่นมีสมรรถนะในการเลี้ยงดูบุตรที่ดี ลดความเครียดในการเลี้ยงดูบุตรของมารดาวัยรุ่นได้ โดยองค์ความรู้ที่ได้รับจากการศึกษาครั้งนี้จะเป็นแนวทางในการพัฒนาระบบการดูแลมารดาวัยรุ่น อันจะก่อให้เกิดประโยชน์ต่อมารดาวัยรุ่นให้สามารถเลี้ยงดูบุตรได้อย่างมีประสิทธิภาพ

4. ทบทวนทฤษฎีและงานวิจัยที่เกี่ยวข้อง (Review literature)

ผู้วิจัยได้ศึกษาเอกสารและงานวิจัยที่เกี่ยวข้อง เพื่อเป็นแนวทางในการศึกษา ดังนี้

1. การตั้งครรภ์ในวัยรุ่น

การตั้งครรภ์ในวัยรุ่น (Adolescent pregnancy) หมายถึง การตั้งครรภ์ในสตรีที่อายุระหว่าง 10-19 ปี โดยนับอายุ ณ เวลาที่คลอดบุตร สามารถแบ่งได้เป็น Adolescent pregnancy คือ การตั้งครรภ์ในสตรีที่อายุระหว่าง 15-19 ปี และ Younger adolescent pregnancy คือ การตั้งครรภ์ในสตรีที่อายุระหว่าง 10-14 ปี

<u>ผลกระทบของการตั้งครรภ์วัยรุ่น</u>

1. ผลกระทบทางด้านร่างกาย ร่างกายในวัยรุ่นยังพัฒนาไม่เต็มที่ ซึ่งอาจก่อให้เกิดปัญหาทางสุขภาพได้มากกว่าวัยผู้ใหญ่ นอกจากนี้การตั้งครรภ์ในวัยรุ่นส่วนใหญ่เป็นการตั้งครรภ์ที่ไม่ได้วางแผน อาจทำให้การดูแลทางสุขภาพหรือการปฏิบัติตัวในขณะตั้งครรภ์นั้นไม่เหมาะสม และอาจเกิดภาวะแทรกซ้อนได้ เช่น ภาวะโลหิตจาง น้ำหนักตัวขึ้นน้อยในขณะตั้งครรภ์ ภาวะความดันโลหิตสูงขณะตั้งครรภ์ การคลอดยาก การคลอดก่อนกำหนด การตกเลือดหลังคลอด น้ำหนักตัวของทารกแรกเกิดน้อย การแท้งบุตรหรือการตายคลอดของทารกแรกเกิด (Althabe et al., 2015; Ogawa et al., 2019)

2. ผลกระทบทางด้านจิตใจ ปัญหาทางสุขภาพจิตเป็นปัญหาที่พบได้บ่อยในหญิงตั้งครรภ์วัยรุ่น หญิงตั้งครรภ์กลุ่มนี้จะเกิดความรู้สึกอับอาย รู้สึกผิดบาปจากการตั้งครรภ์ที่ไม่พร้อม ไม่เป็นที่ยอมรับของครอบครัวหรือสังคม ก่อให้เกิดความ วิตกกังวล ความเครียด และอาจพัฒนาเป็นภาวะซึมเศร้าได้ (Corcoran, 2016; Osok et al., 2018)

3. ผลกระทบต่อครอบครัว ครอบครัวจะเกิดความเครียด โกรธ ผิดหวังในสตรีตั้งครรภ์วัยรุ่น และการถูกตีตราทางสังคม เนื่องจากการตั้งครรภ์ในวัยรุ่นยังไม่เป็นที่ยอมรับในสังคม นอกจากนี้ยังเพิ่มภาระในการดูแลสตรีตั้งครรภ์วัยรุ่นและบุตรที่จะเกิดมาอีกด้วย (จารีศรี, 2561)

4. ผลกระทบต่อสังคมและเศรษฐกิจ ปัญหาทางสังคมที่สำคัญของการตั้งครรภ์วัยรุ่นที่สำคัญคือการทำแท้งที่ผิดกฎหมาย ซึ่งก่อให้เกิดผลกระทบทางสุขภาพที่รุนแรงตามมา เช่น การตกเลือด การติดเชื้อ ฯลฯ นอกจากนี้อาจเกิดปัญหาการทอดทิ้งบุตร หรือการเลี้ยงดูบุตรที่ไม่มีประสิทธิภาพ ทำให้ประเทศชาติได้บุคลากรที่ด้อยคุณภาพหรือกลายเป็นปัญหาของสังคมตามมา รัฐบาลต้องสูญเสียค่าใช้จ่ายในการดูแลวัยรุ่นที่ตั้งครรภ์ ไม่ว่าจะเป็น การรักษาพยาบาลและสวัสดิการทางสังคมอื่น ๆ (จารีศรี, 2561)

INSTITUTION REVIEW BOARD
Queen Savang Vadhana Memorial Hospital
IRB No. ...01........... /...........2566
Date of Approval - 6 ก.พ. 2566

2. แนวคิดบทบาทการเป็นมารดา

2.1 แนวคิดเกี่ยวกับบทบาทการเป็นมารดา

<u>ทฤษฎีเกี่ยวกับการดำรงบทบาทมารดา</u>

ในระยะแรก รูบิน (Rubin, 1975) ได้พัฒนา ทฤษฎีเอกลักษณ์การเป็นมารดา (theory of maternal identity) ประกอบด้วยกระบวนการดังนี้

1. การจำลองแบบ (replication) โดยสตรีจะมีการเลียนแบบ (mimicry) จากมารดาของตนเองหรือมารดาอื่น ๆ
2. การจินตนาการ (fantasy) โดยที่สตรีจะจิตนาการเกี่ยวกับการแสดงบทบาทมารดาของตนเองในอนาคต ว่าจะปฏิบัติต่อบุตรอย่างไร
3. การเลือกแบบอย่างหรือการปฏิเสธบทบาท (dedifferentiation) สตรีมีการเรียนรู้บทบาทมารดา เริ่มแสวงหาแบบอย่างของบทบาทมารดาจากบุคคลใกล้ชิด แล้วตัดสินเลือกแบบอย่างที่เหมาะสมกับตนเองหรือปฏิเสธบทบาทที่คิดว่าไม่เหมาะสม
4. การแสดงเอกลักษณ์การเป็นมารดา (identity) สามารถค้นหารูปแบบบทบาทที่เหมาะสมกับตนเองได้ ยอมรับบทบาทใหม่ มีความพึงพอใจ และแสดงบทบาทมารดาได้ดี

ต่อมาในปี 1985 เมอร์เซอร์ได้พัฒนาแนวคิดของรูบิน โดยอธิบายบทบาทมารดานั้นพัฒนาอย่างต่อเนื่องตั้งแต่ระยะตั้งครรภ์จนถึงหลังคลอด และมีความเป็นพลวัตร ประกอบด้วย 4 ระยะ (Mercer, 2004) ดังนี้

1. ระยะของความมุ่งมั่น ความผูกพัน และการเตรียมเข้าสู่บทบาท (commitment, attachment, preparation) เกิดในระยะตั้งครรภ์ ซึ่งเป็นช่วงที่สตรีมีการเตรียมพร้อมต่อบทบาทมารดา ดูแลสุขภาพตนเอง แสวงหาข้อมูลเกี่ยวกับการปฏิบัติตัวขณะตั้งครรภ์ พัฒนาความรักใคร่ผูกพันกับทารกในครรภ์ และเรียนรู้บทบาทมารดา
2. ระยะของการเพิ่มความผูกพัน การเรียนรู้ และร่างกายฟื้นคืนสู่ภาวะปกติ (acquaintance, learning, physical restoration) เริ่มในระยะทารกคลอด จนถึง 6 สัปดาห์หลังคลอด เป็นระยะที่สตรีแสดงบทบาทมารดาตามคำแนะนำและความคาดหวังของบุคคลอื่น ๆ
3. ระยะพัฒนาบทบาทให้เป็นรูปแบบใหม่ (moving toward a new normal) เริ่มตั้งแต่ 2 สัปดาห์ ถึง 4 เดือนหลังคลอด โดยจะเริ่มแสดงบทบาทมารดาตามลักษณะของตนเองมากขึ้น อาศัยประสบการณ์ การเรียนรู้ที่ผ่านมา ซึ่งเป็นรูปแบบที่มีลักษณะเฉพาะของตนเอง
4. ระยะบรรลุซึ่งเอกลักษณ์การเป็นมารดา (achievement of the maternal identity) เริ่มหลังคลอด 4 เดือนไปแล้ว มารดาเกิดความมั่นใจ มีความสามารถในการเลี้ยงดูบุตร และมีความพึงพอใจ มีความสุขในการที่ได้แสดงบทบาทมารดา

ความสำเร็จในการดำรงบทบาทมารดาสามารถพิจารณาได้จาก 3 องค์ประกอบ ดังนี้ 1) การมีสัมพันธภาพที่ดีกับบุตร (attachment to child) 2) การมีความมั่นใจ หรือมีสมรรถนะในการแสดงบทบาทมารดา (confidence/ competence in role) และ 3) ความพึงพอใจในบทบาทมารดา (gratification/ satisfaction)

2.2 ความเครียดในการเลี้ยงดูบุตร (parental stress)

ความเครียด เป็นอาการที่เกิดจากปฏิกิริยาการตอบสนองของร่างกาย ที่เกิดจากสิ่งเร้าที่มาคุกคาม (stressor) ส่งผลให้เกิดความไม่สบายใจ กังวล สับสน วิตกกังวล และเกิดความไม่สมดุล

ความเครียดในการเลี้ยงดูบุตร (parental stress) เป็นปฏิกิริยาการตอบสนองทางอารมณ์ จากสถานการณ์ต่าง ๆในการเลี้ยงดูบุตรที่เข้ามากระทบ การเลี้ยงดูบุตรเป็นบทบาทใหม่ เป็นระยะแห่งการเรียนรู้วิธีในการเลี้ยงดูบุตร การสร้างสัมพันธภาพกับบุตร รวมไปถึงการเรียนรู้พฤติกรรมของบุตร หากมารดาวัยรุ่นไม่สามารถทำหน้าที่ในการเลี้ยงดูบุตรได้ตามความคาดหวังของตนเองและบุคคลรอบข้าง อาจเกิดความรู้สึกไม่สบายใจ วิตกกังวล ซึ่งจะส่งผลต่อการปรับตัวในการทำหน้าที่เลี้ยงดูบุตรของมารดาวัยรุ่น (สุริยา, รุ่งฤดี และอาภรณ์, 2563)

INSTITUTION REVIEW BOARD
Queen Savang Vadhana Memorial Hospital
IRB No. ....01........... /........2566................
Date of Approval ....- 6 ก.พ. 2566....

Abidin (1995) ได้อธิบายเกี่ยวกับความเครียดในการเลี้ยงดูบุตรว่า หากบิดามารดาไม่สามารถจัดการเกี่ยวกับการเลี้ยงดูบุตรได้ ไม่สามารถตอบสนองความต้องการของบุตรได้อย่างเหมาะสมตามความคาดหวังของตนเองและบุคคลรอบข้าง บิดามารดาจะรู้สึกล้มเหลวกับการทำหน้าที่ในการเป็นบิดามารดาที่ดีได้ (dysfunctional parenting) จากแนวคิดเบื้องต้นดังกล่าว จึงได้พัฒนากรอบแนวคิดในการสร้างแบบสอบถามความเครียดในการเลี้ยงดูบุตร (Theoretical model for the parenting stress index; PSI) เพื่ออธิบายถึงความสัมพันธ์ระหว่างความเครียดในการเลี้ยงดูบุตรกับพฤติกรรมในการเลี้ยงดูบุตร และผลลัพธ์ด้านบุตร ประกอบด้วย 3 องค์ประกอบ ดังนี้

1) ความเครียดจากการทำหน้าที่ในการเลี้ยงดูบุตร (parental distress) หมายถึง ความทุกข์หรือความลำบากใจในการทำหน้าที่ในการเลี้ยงดูบุตร
2) ความเครียดจากการมีปฏิสัมพันธ์ที่ไม่ดีระหว่างมารดาและบุตร (parental – child dysfunction interaction) หมายถึงการรับรู้ของมารดาที่บุตรของตนเองไม่เป็นไปตามที่คาดหวัง และปฏิสัมพันธ์ที่เกิดขึ้นระหว่างมารดาและบุตร ไม่ทำให้เกิดความรู้สึกในการเป็นมารดา และเกิดประสบการณ์ในด้านลบของชีวิต
3) ความเครียดจากพื้นฐานอารมณ์ของบุตร (difficult child) หมายถึง การรับรู้เกี่ยวกับพฤติกรรมหรือลักษณะของบุตรที่ทำให้รู้สึกว่าง่ายหรือยากต่อการจัดการในการเลี้ยงดูบุตร

ซึ่งแสดงความสัมพันธ์ดังกล่าว ดังภาพกรอบแนวคิด

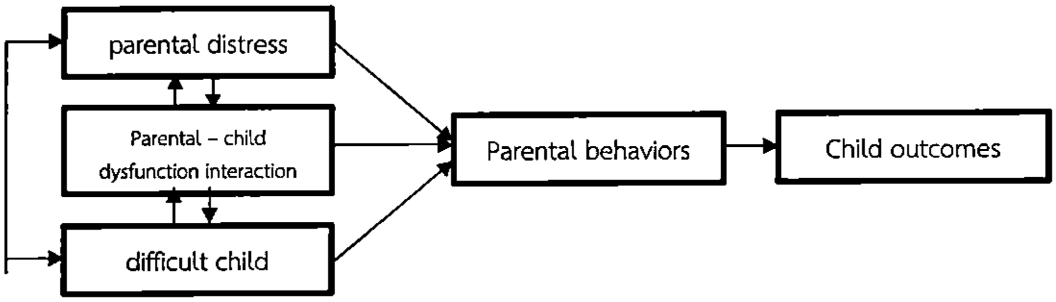

รูปภาพที่ 2: กรอบแนวคิดของแบบสอบถามความเครียดในการเลี้ยงดูบุตร

ที่มา: Abidin, R. R. (1995). Parenting stress index Manual (3rd ed.). Odessa, FL Psychological Assessment Resources.

2.3 สมรรถนะในการเลี้ยงดูบุตร (parental competence)

สมรรถนะในการเลี้ยงดูบุตร (parental competence) เป็นการตัดสินความสามารถของบิดามารดาเกี่ยวกับหน้าที่การเลี้ยงดูบุตรที่ได้ปฏิบัติ โดยเทียบกับความคาดหวังของตนเองหรือสังคม เช่น มารดาประเมินว่าตนเองทำหน้าที่เลี้ยงดูบุตรได้ดีตามความคาดหวังหรือไม่ (Vance & Brandon, 2017) โดยการรับรู้ความสามารถในการเลี้ยงดูบุตรเป็นปัจจัยที่มีความสัมพันธ์กับสมรรถนะในการเลี้ยงดูบุตร และแสดงถึงคุณภาพในการเลี้ยงดูบุตร

<u>การประเมินสมรรถนะในการเลี้ยงดูบุตร</u>

การประเมินสมรรถนะในการเลี้ยงดูบุตรจะประเมินเป็นแบบสอบถาม ซึ่งมีชื่อว่า The Parenting Sense of Competence Scale (PSOC) พัฒนาโดย Gibaud-Wallston และ Wandersman ในปี ค.ศ. 1978 มีองค์ประกอบ 2 ด้านได้แก่ การรับรู้ในความสามารถ (efficacy) ซึ่งเป็นมิติที่แสดงถึงการรับรู้เกี่ยวกับหน้าที่ของตนเองในการเลี้ยงดูบุตร การแก้ไขปัญหาต่าง ๆ ที่เกิดจากการเลี้ยงดูบุตร และความพึงพอใจ (satisfaction) ซึ่งเป็นมิติทางด้านอารมณ์ ที่แสดงถึงความวิตกกังวล ความภาคภูมิใจ และการรู้สึกถึงความสำเร็จในการเลี้ยงดูบุตร

3. ปัจจัยที่มีอิทธิพลต่อการทำหน้าที่ของบิดามารดา

แนวคิดสมรรถนะในการทำหน้าที่ของบิดามารดา (A process model of competent parental functioning) ของเบลสกี้ (Belsky, 1984) ซึ่งอธิบายถึงปัจจัยที่มีอิทธิพลต่อการทำหน้าที่ในการเลี้ยงดูบุตรของบิดามารดา โดยปัจจัยด้านผู้เลี้ยงดูเป็นปัจจัยที่มีอิทธิพลมากที่สุด รองลงมาคือปัจจัยด้านบริบททางสังคมหรือสิ่งแวดล้อม และปัจจัยด้านทารกมีอิทธิพลน้อยที่สุด มีรายละเอียดดังนี้

INSTITUTION REVIEW BOARD
Queen Savang Vadhana Memorial Hospital
[illegible] No. 01 / 2566
Date of Approval - 6 ก.พ. 2566

1. ปัจจัยด้านมารดา

1.1 อายุ เป็นสิ่งที่บ่งบอกถึงการก้าวเข้าสู่วัยต่าง ๆ เช่น การเป็นวัยรุ่น การเป็นวัยผู้ใหญ่ ซึ่งสะท้อนให้เห็นถึงความพร้อมของบุคคล ไม่ว่าจะเป็นทางด้านร่างกาย จิตใจ และสังคม มารดาวัยผู้ใหญ่จะมีความสามารถในการเรียนรู้การคิดวิเคราะห์ และจัดการแก้ไขปัญหาได้ดีกว่า ซึ่งจะทำให้มารดาวัยผู้ใหญ่นั้นรู้สึกว่าตนเองสามารถเลี้ยงดูบุตรได้อย่างมีประสิทธิภาพ (Shrooti et al., 2016)

1.2 สถานภาพสมรส มารดาเลี้ยงเดี่ยวนั้น จะต้องรับภาระในการเลี้ยงดูบุตรคนเดียว ทำให้มารดาเกิดความเครียด หรือมีปัญหาทางด้านจิตใจได้มากกว่า

1.3 สถานะทางเศรษฐกิจ การที่มารดาหรือครอบครัวมีรายได้ที่เพียงพอ จะทำให้รู้สึกมีความมั่นคงในด้านการเงิน จะรู้สึกว่าตนเองนั้นมีสมรรถนะในการเลี้ยงดูบุตรที่มีประสิทธิภาพ แต่มารดาที่มีสถานภาพทางเศรษฐกิจที่ไม่ดี อาจเกิดความเครียดจากภาระทางการเงินที่มากขึ้น และทำให้สมรรถนะในเลี้ยงดูบุตรต่ำได้ (Shrooti et al., 2016)

1.4 ระดับการศึกษา ระดับการศึกษาสูงจะมีความสามารถในการเรียนรู้และการทำความเข้าใจการรับรู้ข้อมูลข่าวสารจากแหล่งต่าง ๆ รวมทั้งความสามารถในการปรับตัวและจัดการปัญหาต่อปัญหาหรือสถานการณ์ต่าง ๆ ได้ดีกว่า (Shrooti et al., 2016)

1.5 จำนวนครั้งของการตั้งครรภ์ เกี่ยวข้องกับการมีประสบการณ์และทักษะในการเลี้ยงดูบุตร การที่มารดาประสบความสำเร็จกับประสบการณ์การเลี้ยงดูบุตรในครั้งก่อนๆ จะส่งผลให้การเลี้ยงดูบุตรในปัจจุบันมีประสิทธิภาพมากขึ้น (Barabach et al., 2017)

1.6 ภาวะสุขภาพทางกายของมารดา การที่มารดามีโรคประจำตัว การเจ็บป่วย หรือภาวะแทรกซ้อนทางสูติศาสตร์นั้น จะทำให้มารดาต้องมีการดูแลสุขภาพของตนเองมากกว่ามารดาที่ไม่มีโรคประจำตัว ซึ่งอาจทำให้ประสิทธิภาพในการเลี้ยงดูบุตรลดลง (Maehara et al., 2016)

1.7 ความพร้อมในการตั้งครรภ์หรือการมีบุตร มารดาที่มีความพร้อมในการตั้งครรภ์หรือการมีบุตร จะมีการเตรียมตัวต่อการตั้งครรภ์หรือการเลี้ยงดูบุตร (Shrooti et al., 2016)

1.8 การรับรู้คุณค่าในตนเอง (self-esteem) การที่มารดามีการรับรู้คุณค่าในตนเองสูง จะสามารถปรับตัวหรือเผชิญสถานการณ์ต่าง ๆ ได้อย่างราบรื่น ซึ่งจะส่งผลให้มารดาทำหน้าที่เลี้ยงดูบุตรได้ดี (Shrooti et al., 2016)

1.9 ภาวะสุขภาพทางจิตของมารดา ความเครียด ความกังวลกับการเลี้ยงดูบุตร หรือภาวะซึมเศร้า อาจทำให้ไม่มีประสิทธิภาพในการเลี้ยงดูบุตร (Angley et al., 2015; Shorey et al., 2015)

1.10 การรับรู้สมรรถนะในตนเอง (self-efficacy) หากมารดามีการรับรู้สมรรถนะแห่งตนเองสูง จะมีความมั่นใจว่าสามารถเลี้ยงดูบุตรได้ และส่งผลให้มารดามีแนวโน้มในการปฏิบัติการเลี้ยงดูบุตรได้สำเร็จ

2. ปัจจัยด้านสิ่งแวดล้อม

2.1 การสนับสนุนทางสังคม สามารถส่งเสริมให้มารดามีสมรรถนะในการเลี้ยงดูบุตร โดยการให้การช่วยเหลือ ให้การสนับสนุนด้านทรัพยากร ข้อมูลข่าวสาร รวมทั้งการให้แรงเสริม หรือกำลังใจในการเลี้ยงดูบุตร (Brown et al., 2016)

2.2 สัมพันธภาพกับคู่สมรส การที่มารดามีสัมพันธภาพที่ดีกับสามี มีความรัก ความห่วงใยต่อกัน มารดาจะได้รับการสนับสนุนที่ดีจากสามี (Kershaw, 2014)

3. ปัจจัยด้านบุตร

3.1 ภาวะสุขภาพของบุตร ทารกที่มีภาวะแทรกซ้อนหรือการเจ็บป่วย เช่น ทารกมีน้ำหนักแรกคลอดน้อย ทารกเกิดก่อนกำหนด หรือทารกมีความพิการแต่กำเนิด มารดาจะเครียดกับภาวะสุขภาพของทารกอาจส่งผลต่อสมรรถนะในการเลี้ยงดูบุตร (Korja, Latva, & Lehtonen, 2012)

3.2 พื้นฐานอารมณ์ของบุตร การที่บุตรนั้นร้องไห้ง่าย ปลอบโยนได้ยาก มารดาจะรับรู้ว่ามีความยากลำบากในการจัดการกับอารมณ์ของบุตร รับรู้ได้ว่าการเป็นมารดานั้นมีความยากลำบากและทำให้มารดารู้สึกถึงประสิทธิภาพและสมรรถนะของตนในการเลี้ยงดูบุตรว่ามีน้อย (Ponomartchouk & Bouchard, 2015)

INSTITUTION REVIEW BOARD
Queen Savang Vadhana Memorial Hospital
IRB No. ....01........ /....2566............
Date of Approval ....- 6 ก.พ. 2566....

4. การใช้ Line official account ในการดูแลสุขภาพ

Line official account หมายถึง ฟีเจอร์หนึ่งของแอพลิเคชั่นไลน์ ซึ่งถูกพัฒนามาให้เป็นบัญชีธุรกิจหรือบริษัท เพื่อส่งเสริมกิจกรรมทางธุรกิจหรือการส่งข้อมูลข่าวสารต่าง ๆ ในวงกว้างมากขึ้น สามารถที่จะเผยแพร่รูปแบบของสารที่หลากหลาย (multimedia) เช่น ข้อความ รูปภาพ โปสเตอร์ วิดีโอ หรือการประชาสัมพันธ์ให้กับผู้ติดตามได้สะดวก นอกจากนี้ยังเป็นช่องทางในการติดต่อสื่อสาร โดยผู้ที่ติดตามสามารถส่งข้อความกลับมา เพื่อพูดคุยหรือการปรึกษาได้ โดยที่บุคคลอื่น ๆ ใน line official account จะไม่เห็นข้อความดังกล่าว

จุดเด่นของแอพลิเคชั่นไลน์ เป็นแอพลิเคชั่นที่สามารถสร้างกลุ่มสนทนา โดยมีการเชิญและตอบรับการเข้ากลุ่ม ซึ่งกลุ่มไลน์ส่วนใหญ่จะเป็นไปในลักษณะของสมาชิกที่มีประสบการณ์เดียวกัน หรือมีลักษณะบางอย่างที่มีความคล้ายคลึงกัน สามารถส่งเสริมให้การติดต่อสื่อสารสะดวกมากขึ้น เพราะการส่งข้อความออกไป 1 ครั้ง บุคคลในกลุ่มการสนทนานั้นจะรับรู้การสื่อสารทั้งหมด แอพลิเคชั่นรองรับไฟล์ได้อย่างหลากหลาย ไม่ว่าจะเป็นรูปแบบข้อความ เสียง รูปภาพ วิดีโอ สติ๊กเกอร์ โปสเตอร์ ไฟล์เอกสาร ซึ่งสามารถเลือกรูปแบบให้มีความเหมาะสมกับกลุ่มเป้าหมายในการสื่อสารได้ รวมทั้งประหยัดค่าใช้จ่ายในการสนทนาทางโทรศัพท์ ซึ่งสามารถสนทนาด้วยเสียง (voice call) แทน ทำให้ไม่เสียค่าใช้จ่ายนอกเหนือจากแพ็คเกจอินเตอร์เน็ต (วิสา การบุญ, 2560)

ปัจจุบันโลกและประเทศไทยได้ก้าวเข้าสู่ยุค Digital 4.0 เป็นยุคที่มีความฉลาดของเทคโนโลยี ในวงการสุขภาพก็ได้เกิดการเปลี่ยนแปลงในการเข้าถึงหรือการดูแลสุขภาพของผู้รับบริการ ในรูปแบบของ Digital health มากขึ้นผ่านรูปแบบต่าง ๆ ไม่ว่าจะเป็น แอพลิเคชั่น เว็ปไซต์ ข้อความอิเล็กทรอนิกส์ อุปกรณ์สวมใส่ เซ็นเซอร์การตรวจวัดการทำงานของร่างกาย เป็นต้น เพื่อพัฒนาการบริการทางสุขภาพให้มีประสิทธิภาพมากขึ้น แอพลิเคชั่นไลน์จึงเป็นช่องทางที่น่าสนใจและเข้าถึงกลุ่มวัยรุ่นได้ดี โดย line official account เป็นบัญชีที่สร้างขึ้นเพื่อการส่งข้อมูลข่าวสารต่าง ๆ ในวงกว้าง สามารถที่จะเผยแพร่ข้อความ รูปภาพ วิดีโอ หรือการประชาสัมพันธ์ให้กับผู้ติดตามได้สะดวก นอกจากนี้ยังเป็นช่องทางในการติดต่อสื่อสาร โดยผู้ที่ติดตามสามารถส่งข้อความกลับมาเพื่อพูดคุยหรือปรึกษาได้ โดยที่บุคคลอื่น ๆ ใน line official account จะไม่เห็นข้อความดังกล่าว ซึ่งเป็นช่องทางในการติดต่อสื่อสารที่มีความเป็นส่วนตัว และไม่มีค่าใช้จ่ายเพิ่มเติมจากแพ็คเกจอินเตอร์เน็ต ดังนั้นการใช้ line official account มาเป็นสื่อกลางในการเผยแพร่ความรู้ จะเป็นประโยชน์ในการติดต่อสื่อสาร และเป็นช่องทางในการปรึกษาปัญหาของมารดาวัยรุ่นกับบุคลากรทางสุขภาพได้อย่างสะดวกและรวดเร็วยิ่งขึ้น

5. วัตถุประสงค์ของการวิจัย (Objectives)

1. เพื่อสร้างนวัตกรรม line official account “Parent พาเพลิน”
2. เพื่อเปรียบเทียบความเครียดในการเลี้ยงดูบุตรของมารดาวัยรุ่นหลังคลอด ที่ได้รับโปรแกรมส่งเสริมการเลี้ยงดูบุตรร่วมกับการใช้ line official account “Parent พาเพลิน” กับมารดาวัยรุ่นหลังคลอดที่ได้รับการพยาบาลตามปกติ
3. เพื่อเปรียบเทียบสมรรถนะในการเลี้ยงดูบุตรของมารดาวัยรุ่นหลังคลอด ที่ได้รับโปรแกรมส่งเสริมการเลี้ยงดูบุตรร่วมกับการใช้ line official account “Parent พาเพลิน” กับมารดาวัยรุ่นหลังคลอดที่ได้รับการพยาบาลตามปกติ

6. กรอบแนวคิด

ในการศึกษาครั้งนี้ผู้วิจัยใช้กรอบแนวคิดสมรรถนะในการทำหน้าที่ของบิดามารดา (A process model of competent parental functioning) ของเบลสกี้ (Belsky, 1984) อธิบายถึงปัจจัยที่มีอิทธิพลต่อการทำหน้าที่ในการเลี้ยงดูบุตรของบิดามารดา ซึ่งเป็นปัจจัยที่ส่งผลต่อการเลี้ยงดูบุตรที่มีประสิทธิภาพ และส่งผลต่อพัฒนาการของบุตรในอนาคต ประกอบไปด้วย 3 ปัจจัย ได้แก่ 1) ปัจจัยด้านผู้เลี้ยงดู เป็นคุณลักษณะส่วนบุคคลของผู้เลี้ยงดู ซึ่งส่งผลต่อการทำหน้าที่ในการเลี้ยงดูบุตร 2) ปัจจัยด้านบริบททางสังคมหรือสิ่งแวดล้อม เป็นแหล่งความเครียดและการสนับสนุนทางสังคม และ 3) ปัจจัยด้านทารก โดยปัจจัยด้านผู้เลี้ยงดูเป็นปัจจัยที่มีอิทธิพลมากที่สุด รองลงมาคือปัจจัยด้านบริบททางสังคมหรือสิ่งแวดล้อม และปัจจัยด้านทารกมีอิทธิพลน้อยที่สุด

INSTITUTION REVIEW BOARD
Queen Savang Vadhana Memorial Hospital
IRB No. ......01......... /.......2566..........
Date of Approval - 6 ก.พ. 2566

ผู้วิจัยจึงประยุกต์ใช้กรอบแนวคิดสมรรถนะในการทำหน้าที่ของบิดามารดาในการสร้างโปรแกรมการส่งเสริมการเลี้ยงดูบุตรร่วมกับ line official account “Parent พาเพลิน” ซึ่งโปรแกรมจะเป็นปัจจัยด้านบริบททางสังคมหรือสิ่งแวดล้อม พยาบาลเป็นสิ่งแวดล้อมที่สำคัญในการสนับสนุนให้มารดาวัยรุ่นสามารถเลี้ยงดูบุตรได้อย่างมีประสิทธิภาพ โดยเน้นการให้ความรู้และฝึกทักษะการเลี้ยงดูบุตร เช่น สื่อสัญญาณทารกและการตอบสนองต่อความต้องการของทารก การเลี้ยงบุตรด้วยนมมารดา การอาบน้ำบุตร เป็นต้น นอกจากนี้ยังติดตามสอบถามปัญหาและเปิดช่องทางในการให้คำปรึกษาผ่าน line official account ซึ่งจะทำให้มารดาวัยรุ่นมีความเครียดในการเลี้ยงดูบุตรลดลงและมีสมรรถนะในการเลี้ยงดูบุตรเพิ่มขึ้น สำหรับปัจจัยด้านอื่นผู้วิจัยได้นำมาใช้ในการควบคุมตัวแปร ได้แก่ ปัจจัยด้านผู้เลี้ยงดู โดยคำนึงถึงจำนวนครั้งการตั้งครรภ์ ความต้องการมีบุตร สถานภาพการสมรส ประสบการณ์การเลี้ยงดูทารก สถานะทางเศรษฐกิจ และปัจจัยด้านทารกคำนึงถึงภาวะสุขภาพของบุตร สามารถแสดงเป็นรูปภาพกรอบแนวคิดการวิจัยดังนี้

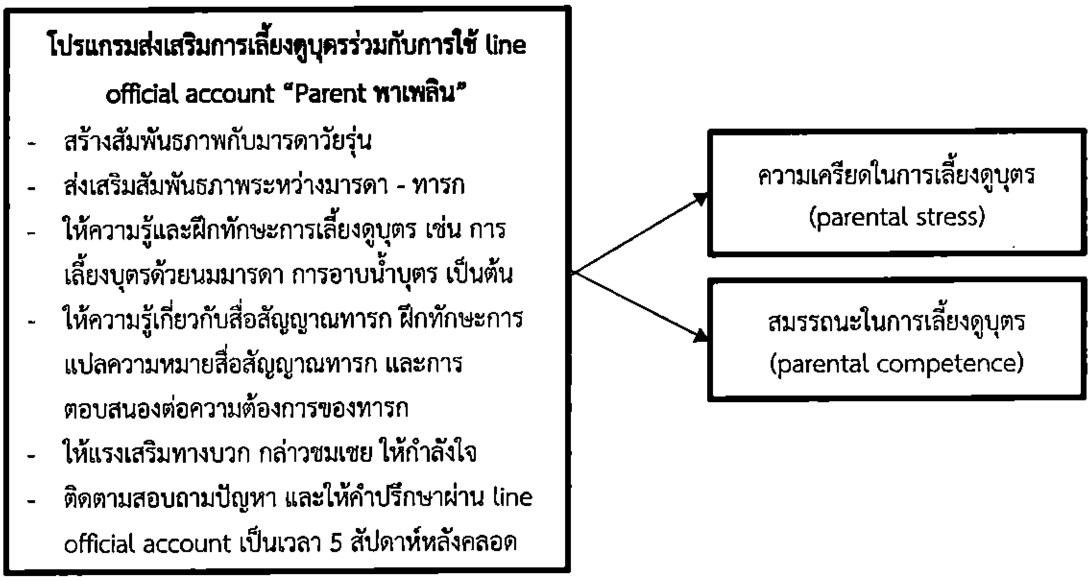

รูปภาพ 1: กรอบแนวคิดการวิจัย

7. สมมติฐานการวิจัย (Research hypothesis)

1. มารดาวัยรุ่นหลังคลอดที่ได้รับโปรแกรมส่งเสริมการเลี้ยงดูบุตรร่วมกับการใช้ line official account “Parent พาเพลิน” มีความเครียดในการเลี้ยงดูบุตร ต่ำกว่ามารดาวัยรุ่นหลังคลอดที่ได้รับการพยาบาลตามปกติ
2. มารดาวัยรุ่นหลังคลอดที่ได้รับโปรแกรมส่งเสริมการเลี้ยงดูบุตรร่วมกับการใช้ line official account “Parent พาเพลิน” มีสมรรถนะในการเลี้ยงดูบุตร สูงกว่ามารดาวัยรุ่นหลังคลอดที่ได้รับการพยาบาลตามปกติ

8. คำสำคัญ (Keywords)

โปรแกรมการเลี้ยงดูบุตร Line Official Account ความเครียดในการเลี้ยงดูบุตร สมรรถนะในการเลี้ยงดูบุตร มารดาวัยรุ่นหลังคลอด

9. รูปแบบการวิจัย (Research Design)

การศึกษานี้เป็นกึ่งทดลอง (quasi-experimental research design) ประเภทการวิจัยแบบสองกลุ่มวัดก่อนและหลังทดลอง (the control-group pretest-posttest design)

10. ระเบียบวิธีการวิจัย (Research methodology)

10.1 ประชากร (Population)

ประชากรในการศึกษาครั้งนี้คือมารดาอายุ 15 - 19 ปี

10.2 กลุ่มตัวอย่าง (Sample)

ประชากรในการศึกษาครั้งนี้คือมารดาอายุ 15 - 19 ปี ที่เข้าพักรักษาตัว ณ หอผู้ป่วยสูติกรรมสามัญ โรงพยาบาลสมเด็จพระบรมราชเทวี ณ ศรีราชา สภากาชาดไทย

INSTITUTION REVIEW BOARD
[illegible]een Savang Vadhana Memorial Hospital
No. 01 / 2566
Date of Approval - 6 ก.พ. 2566

10.3 ขนาดตัวอย่างและการคำนวณ (Sample size estimation)

คำนวณขนาดกลุ่มตัวอย่างด้วยโปรแกรมสำเร็จรูป G Power version 3.1.9.7 โดยคำนวณขนาดอิทธิพลของประชากร (effect size) ซึ่งอ้างอิงจากงานวิจัยที่ใกล้เคียงของ Moudi และคณะ (2020) ที่ศึกษาผลของโปรแกรมการฝึกอบรมเบื้องต้นสำหรับมารดาวัยรุ่นครรภ์แรกต่อสมรรถนะแห่งตนในการเลี้ยงดูบุตรและความผูกพันมารดา - ทารก โดยมีวัตถุประสงค์และผลลัพธ์ในการศึกษาใกล้เคียงกับการศึกษาครั้งนี้ กำหนดค่าอำนาจการทดสอบ (Power of Test; β) = .80 กำหนดระดับนัยสำคัญทางสถิติ .05 และกำหนดขนาดอิทธิพลขนาดใหญ่ = 0.8 สำหรับการทดสอบสมมติฐานด้วยสถิติ independent t-test ได้ขนาดกลุ่มตัวอย่างจำนวนกลุ่มละ 26 ราย ทั้งนี้เพื่อป้องกันการสูญหายระหว่างการศึกษา ผู้วิจัยได้เพิ่มกลุ่มตัวอย่างอีกร้อยละ 10 ดังนั้นในการศึกษาครั้งนี้จึงใช้กลุ่มตัวอย่างในกลุ่มทดลองจำนวน 30 ราย และกลุ่มควบคุมจำนวน 30 ราย รวมจำนวนกลุ่มตัวอย่างทั้งหมด 60 ราย

10.4 เกณฑ์การคัดเลือกอาสาสมัครเข้าร่วมโครงการวิจัย (Inclusion criteria)

คัดเลือกกลุ่มตัวอย่างแบบเฉพาะเจาะจง (purposive sampling) จากประชากรดังกล่าว โดยกำหนดคุณสมบัติของกลุ่มตัวอย่าง (inclusion criteria) ดังนี้

1. มารดาหลังคลอดครบกำหนด 37-42 สัปดาห์
2. มารดาหลังคลอดอายุ 15-19 ปี
3. มารดาไม่มีภาวะแทรกซ้อนหลังคลอดที่รุนแรง เช่น ภาวะตกเลือกหลังคลอด, preeclampsia with severe feature เป็นต้น
4. ทารกไม่มีภาวะแทรกซ้อนที่รุนแรง พิการแต่กำเนิดที่ต้องเข้ารับการรักษาใน NICU หรือต้องแยกจากมารดา
5. มารดาวางแผนเลี้ยงบุตรด้วยตนเองใน 6 สัปดาห์แรกหลังคลอด
6. สามารถอ่าน เขียน และสื่อสารภาษาไทยได้
7. สามารถติดต่อสื่อสารผ่านสมาร์ทโฟนที่มี Line application ได้

เกณฑ์การคัดเลือกอาสาสมัครออกจากโครงการวิจัย (Exclusion criteria)

1. เลี้ยงดูบุตรด้วยตนเองไม่ครบ 6 สัปดาห์หลังคลอด
2. ไม่สามารถเข้าร่วมการวิจัยได้ครบทุกขั้นตอน
3. ต้องการถอนตัวออกจากการวิจัย

10.5 กระบวนการขอความยินยอม (Informed consent process)

ภายหลังจากการได้รับอนุญาตในการเข้าพบจากอาสาสมัครเรียบร้อยแล้ว ผู้วิจัยได้อธิบายวัตถุประสงค์ของการวิจัย ขั้นตอนของการเก็บข้อมูล ประโยชน์ที่ได้รับและความเสี่ยงที่อาจเกิดขึ้นจากการเข้าร่วมโครงการวิจัย และเปิดโอกาสให้อาสาสมัครได้ซักถามข้อสงสัย พร้อมทั้งตอบข้อสงสัยจากอาสาสมัคร โดยอาสาสมัครเป็นผู้ตัดสินใจที่จะเข้าร่วมการวิจัยด้วยตนเอง มีสิทธิปฏิเสธการเข้าร่วมการวิจัย มีสิทธิที่จะปฏิเสธการตอบคำถามในข้อที่ไม่ต้องการตอบ และถอนตัวจากการเข้าร่วมวิจัยได้ตลอดเวลาโดยไม่มีผลกระทบใด ๆ ต่อการบริการที่กลุ่มตัวอย่างจะได้รับ หากมารดาวัยรุ่นมีความประสงค์ที่จะเข้าร่วมโครงการวิจัย จึงให้เซ็นใบยินยอมเข้าร่วมการวิจัย ในกรณีกลุ่มตัวอย่างอายุน้อยกว่า 18 ปี ต้องได้รับความยินยอมจากผู้ปกครองหรือสามีก่อน

10.6 เครื่องมือและการตรวจคุณภาพเครื่องมือ (Instrument)

10.6.1 เครื่องมือที่ใช้ในการทดลอง

1. โปรแกรมส่งเสริมการเลี้ยงดูบุตร เป็นชุดกิจกรรมในการส่งเสริมความสามารถในการเลี้ยงดูบุตร โดยมีพื้นฐานตามแนวคิดสมรรถนะในการทำหน้าที่ของบิดามารดา (A process model of competent parental functioning) ของเบลสกี้ (Belsky, 1984) ประกอบด้วยกิจกรรมดังนี้

- ครั้งที่ 1 ระยะ 24 – 48 ชั่วโมงหลังคลอด ผู้วิจัยสร้างสัมพันธภาพ แนะนำตัวเพื่อสร้างสัมพันธภาพ จากนั้นผู้วิจัยส่งเสริมสัมพันธภาพระหว่างมารดา - ทารก ให้ความรู้และฝึกทักษะรายบุคคลเกี่ยวกับสื่อสัญญาณทารกและการตอบสนองต่อสื่อสัญญาณทารก การดูแลบุตร การเลี้ยงบุตรด้วยนมมารดา การอาบน้ำบุตร และการสังเกตอาการผิดปกติของทารก โดยใช้สื่อผ่าน line official account "Parent พาเพลิน"

INSTITUTION REVIEW BOARD
[illegible]een Savang Vadhana Memorial Hospital
[illegible] No. 01 / 2566
Date of Approval - 6 ก.พ. 2566

ใช้เวลา 60 – 90 นาที

- ครั้งที่ 2 ระยะ 48 – 72 ชั่วโมงหลังคลอด ทบทวนความรู้ ฝึกทักษะเพิ่มเติม เพื่อส่งเสริมความมั่นใจของมารดา หากมารดาวัยรุ่นยังขาดความมั่นใจ ดูแลให้ความช่วยเหลือรายบุคคลตามปัญหาที่เกิดขึ้นในแต่ละบุคคล ใช้เวลา 60 นาที

- ครั้งที่ 3 ติดตามภายหลังการจำหน่ายออกจากโรงพยาบาล โดยติดตามทุกสัปดาห์ เป็นเวลา 5 สัปดาห์ โดยสอบถามปัญหาและให้คำปรึกษาผ่าน line official account

2. Line official account “Parent พาเพลิน” เป็นแอพลิเคชั่นสำหรับการสนทนาในรูปแบบของข้อความ รูปภาพ วิดีโอในวงกว้าง และมีช่องทางในการติดต่อสื่อสาร เพื่อให้มารดาวัยรุ่นสามารถส่งข้อความกลับมาพูดคุยหรือปรึกษาได้ โดยที่บุคคลอื่น ๆ ใน line official account จะไม่เห็นข้อความดังกล่าว โดยภายใน Line official account “Parent พาเพลิน” จะมีเนื้อหาเกี่ยวกับสื่อสัญญาณทารกและการตอบสนองต่อสื่อสัญญาณทารก การดูแลบุตร การเลี้ยงบุตรด้วยนมมารดา การอาบน้ำบุตร และการสังเกตอาการผิดปกติของทารก ในรูปแบบของ infographics, e-book หรือวิดีทัศน์

10.6.2 แบบสอบถาม/แบบประเมิน (Questionnaire or Assessment form)

1. แบบสัมภาษณ์ข้อมูลส่วนบุคคล เป็นเครื่องมือที่ผู้วิจัยสร้างขึ้นเอง มี 2 ส่วน ดังนี้ ส่วนที่ 1 ข้อมูลทั่วไป ประกอบด้วย อายุมารดา สถานภาพสมรส ระดับการศึกษา การประกอบอาชีพ รายได้ครอบครัว ความเพียงพอของรายรับกับรายจ่าย ลักษณะครอบครัว ความต้องการมีบุตร การวางแผนศึกษาต่อ บุคคลที่ช่วยเหลือในการเลี้ยงดูบุตร ประสบการณ์การเลี้ยงดูเด็กทารก ส่วนที่ 2 ข้อมูลทางสูติกรรม ประกอบด้วย จำนวนครั้งของการตั้งครรภ์ วิธีการคลอด การวางแผนคุมกำเนิด คะแนนภาวะซึมเศร้าของมารดาในระยะหลังคลอด และคะแนน APGAR ของบุตร หากข้อมูลใดที่กลุ่มตัวอย่างไม่สามารถตอบได้ ผู้วิจัยจะขออนุญาตเก็บรวบรวมข้อมูลจากแฟ้มประวัติ

2. แบบบันทึกปัญหาและการให้คำแนะนำภายหลังการจำหน่าย ผู้วิจัยสร้างขึ้นเพื่อบันทึกการติดตามภายหลังการจำหน่ายออกจากโรงพยาบาลทาง line official account ประกอบด้วย ครั้งที่ วันที่ ปัญหาที่พบ การดูแลหรือการให้คำแนะนำ

3. แบบประเมินภาวะซึมเศร้าหลังคลอด ใช้ Edinburgh Postnatal Depression Scale (EPDS) ฉบับภาษาไทยของกมลรัตน์ วัชราภรณ์และคณะ (2546) แบบประเมินใช้ประเมินความรู้สึก อารมณ์และพฤติกรรมที่เกิดขึ้นในช่วง 1 สัปดาห์ที่ผ่านมา มีข้อคำถามจำนวน 10 ข้อ ลักษณะแบบสอบถามเป็นมาตราส่วนประมาณค่า (likert scale) 4 ระดับ ตั้งแต่ 0-3 คะแนน คะแนนรวมมีค่าต่ำสุดเท่ากับ 0 คะแนน และค่าสูงสุดเท่ากับ 30 คะแนน ใช้เกณฑ์จุดตัดคะแนนเท่ากับ 11 คะแนนขึ้นไป หรือมีคะแนนในข้อสุดท้าย ซึ่งเป็นข้อคำถามเกี่ยวกับความคิดในการฆ่าตัวตาย ในการคัดกรองภาวะซึมเศร้าของกลุ่มตัวอย่างเพื่อให้การดูแลช่วยเหลือทางด้านอารมณ์และจิตใจ

4. แบบประเมินความเครียดในการเลี้ยงดูบุตร ใช้แบบประเมิน Parenting Stress Index Fourth Edition Short Form (PSI-4-SF) ฉบับภาษาไทยของสุนทรี ศรีโกไสย, พัชรี หมื่นชัย, ชฎาพร คำฟู, ศิริวรรณ ทวีวัฒนปรีชา และรัตนา สายพานิชย์ (2563) ซึ่งแปลเป็นภาษาไทยด้วยวิธีแปลย้อนกลับจากเครื่องมือต้นฉบับของ Abidin (1995) แบบประเมินมี 36 ข้อ ลักษณะเป็นมาตราส่วนประมาณค่า (likert scale) 5 ระดับ แต่ในการศึกษามีการปรับลดจำนวนข้อเหลือเพียง 15 ข้อ เพื่อให้สอดคล้องกับบริบทในการศึกษาตามอายุของทารก 6 สัปดาห์หลังคลอด คะแนนรวมเท่ากับ 14 – 75 คะแนน คะแนนต่ำ หมายถึง มารดาวัยรุ่นมีความเครียดในการเลี้ยงดูบุตรต่ำ คะแนนสูง หมายถึง มารดาวัยรุ่นมีความเครียดในการเลี้ยงดูบุตรสูง

5. แบบประเมินสมรรถนะในการเลี้ยงดูบุตร โดยใช้ The Parenting Sense of Competence Scale (PSOC) ฉบับภาษาไทยของสุนีย์ กลีบปาน, วรรณา พาหุวัฒนกร, ฉวีวรรณ อยู่สำราญ และวิมลนันท์ พุฒิวณิชพงศ์ (2562) ภายใต้ลิขสิทธิ์ของมหาวิทยาลัยมหิดล ซึ่งได้แปลด้วยวิธีแปลย้อนกลับจากเครื่องมือต้นฉบับของ Jhonston & Mash (1989) แบบสอบถามมีทั้งหมด 16 คำถาม ลักษณะเป็นมาตราส่วนประมาณค่า (likert scale) 6 ระดับ คะแนนรวมเท่ากับ 16-96 คะแนน คะแนนต่ำหมายถึง มารดาวัยรุ่นมีสมรรถนะในการเลี้ยงดูบุตรต่ำ คะแนนสูงหมายถึง มารดาวัยรุ่นมีสมรรถนะในการเลี้ยงดูบุตรสูง

การตรวจสอบคุณภาพเครื่องมือวิจัย

1. การหาความเที่ยงตรงของเครื่องมือ (validity)

ผู้วิจัยนำเครื่องมือวิจัย เสนอต่อผู้ทรงคุณวุฒิทั้งหมด 3 ท่าน เป็นอาจารย์พยาบาลด้านการพยาบาลมารดาทารก และการผดุงครรภ์ 2 ท่าน และพยาบาลผู้เชี่ยวชาญด้านการพยาบาลมารดาหลังคลอด 1 ท่าน เพื่อพิจารณาตรวจสอบความเที่ยงตรงของเนื้อหา ความถูกต้องเหมาะสมของภาษา และภาพประกอบที่ใช้ ความสอดคล้องกับวัตถุประสงค์ และความครอบคลุมเนื้อหา รวมทั้งให้ข้อเสนอแนะเพื่อปรับปรุงแก้ไข นำมาวิเคราะห์ค่าดัชนีความตรงเชิงเนื้อหา (content validity index; CVI) และนำมาปรับปรุงแก้ไขตามข้อเสนอแนะของผู้ทรงคุณวุฒิ โดยมีรายละเอียดดังนี้

- สื่อใน line official account "Parent พาเพลิน" ค่า CVI = 1
- แบบสัมภาษณ์ข้อมูลส่วนบุคคล ค่า CVI = 0.93
- แบบบันทึกปัญหาและการให้คำแนะนำหลังการจำหน่าย ค่า CVI = 1
- แบบประเมินภาวะซึมเศร้าหลังคลอด ค่า CVI = 0.7
- แบบประเมินความเครียดในการเลี้ยงดูบุตร ค่า CVI = 0.92
- แบบประเมินสมรรถนะในการเลี้ยงดูบุตร ค่า CVI = 0.75

2. การหาความเชื่อมั่นของเครื่องมือ (reliability)

ผู้วิจัยนำ line official account "Parent พาเพลิน", แบบประเมินความเครียดในการเลี้ยงดูบุตร และแบบประเมินสมรรถนะในการเลี้ยงดูบุตร ไปทดสอบกับมารดาวัยรุ่นหลังคลอดที่มีลักษณะใกล้เคียงกับกลุ่มตัวอย่าง จำนวน 30 ราย ที่โรงพยาบาลสมเด็จพระบรมราชเทวี ณ ศรีราชา สภากาชาดไทย ซึ่งได้ดำเนินการหลังจากได้รับการรับรองจากคณะกรรมการจริยธรรมการวิจัยของโรงพยาบาลสมเด็จพระบรมราชเทวี ณ ศรีราชา สภากาชาดไทย ภายหลังจากนั้นนำมาหาค่าความเชื่อมั่น (reliability) ด้วยวิธีสัมประสิทธิ์แอลฟาของครอนบาค (cronbach's alpha coefficient)

11. การรวบรวมข้อมูล (Data Collection)

11.1 ขั้นตอนนำอาสาสมัครเข้าทำการวิจัย

ผู้วิจัยขอความร่วมมือพยาบาลหอผู้ป่วยหลังคลอดสามัญในการคัดเลือกกลุ่มตัวอย่างที่มีคุณสมบัติตรงตามเกณฑ์ที่กำหนด โดยพยาบาลหอผู้ป่วยหลังคลอดสามัญขออนุญาตให้ผู้เข้าวิจัยเข้าพบ เมื่ออาสาสมัครอนุญาต ผู้วิจัยจึงเข้าพบอาสาสมัครเพื่อชี้แจงรายละเอียดของโครงการวิจัย และสอบถามความสมัครใจในการเข้าร่วมการวิจัยครั้งนี้

11.2 วิธีการเก็บรวบรวมข้อมูล

1. เสนอโครงการวิจัยต่อคณะกรรมการจริยธรรมการวิจัยของโรงพยาบาลสมเด็จพระบรมราชเทวี ณ ศรีราชา สภากาชาดไทย เพื่อขออนุญาตในการเก็บรวบรวมข้อมูล
2. หลังจากได้รับอนุมัติ ผู้วิจัยทำหนังสือขออนุญาตเก็บข้อมูล เสนอต่อผู้อำนวยการโรงพยาบาลสมเด็จพระบรมราชเทวี ณ ศรีราชา สภากาชาดไทย ซึ่งจะถูกส่งต่อไปยังฝ่ายการพยาบาล หัวหน้าหอผู้ป่วยที่เกี่ยวข้อง เพื่อขออนุญาตเก็บรวบรวมข้อมูล
3. หลังจากได้รับอนุญาตให้เก็บรวบรวมข้อมูล ผู้วิจัยแนะนำตัวต่อหัวหน้าหอผู้ป่วยที่เกี่ยวข้อง เพื่อชี้แจงรายละเอียดเกี่ยวกับการวิจัย และขอความร่วมมือในการเก็บข้อมูล
4. ผู้วิจัยขอความร่วมมือพยาบาลหอผู้ป่วยหลังคลอดสามัญในการคัดเลือกกลุ่มตัวอย่างที่มีคุณสมบัติตรงตามเกณฑ์ที่กำหนด โดยพยาบาลหอผู้ป่วยหลังคลอดสามัญขออนุญาตให้ผู้เข้าวิจัยเข้าพบ โดยมีขั้นตอนในการเก็บกลุ่มตัวอย่างแต่ละกลุ่ม ดังรูปภาพที่ 3

INSTITUTION REVIEW BOARD
Queen Savang Vadhana Memorial Hospital
IRB No. 01 / 2566
Date of Approval - 6 ก.พ. 2566

มารดาวัยรุ่นที่มีคุณสมบัติตามเกณฑ์ที่กำหนด

คัดเลือกเข้ากลุ่มด้วย Match paired sampling

กลุ่มควบคุม

<u>24 ชั่วโมงหลังคลอด</u> ผู้วิจัยสัมภาษณ์ข้อมูลส่วนบุคคลและให้ทำแบบประเมินความเครียดในการเลี้ยงดูบุตร และแบบประเมินสมรรถนะในการเลี้ยงดูบุตร (Pre-test) ผ่าน Link microsoft forms

การพยาบาลตามปกติ

- <u>ระยะ 24 - 48 ชั่วโมงหลังคลอด</u> Miss mom สอนการเลี้ยงบุตรด้วยนมมารดาใน และสอนอาบน้ำบุตรในเวลา 14.00 น. สำหรับมารดาหลังคลอดบุตรคนแรก สอนการทำความสะอาดสะดือในเวลา 14.00 น. สำหรับมารดาหลังคลอดบุตรคนที่สองขึ้นไป
- <u>ระยะ 48 – 72 ชั่วโมงหลังคลอด</u> เตรียมจำหน่ายออกจากโรงพยาบาล โดยสอนสุขศึกษารายกลุ่มเกี่ยวกับการดูแลตนเองหลังคลอด และหอผู้ป่วยมี line official account สำหรับเป็นช่องทางให้มารดาติดต่อมาปรึกษา โดยจะมีพยาบาลตอบคำถามปัญหาที่มารดาสงสัย

<u>1 สัปดาห์หลังคลอด</u> ทำแบบประเมินภาวะซึมเศร้าหลังคลอด หากมีภาวะซึมเศร้าหลังคลอด ผู้วิจัยจะไม่คัดออกจากการวิจัย แต่ดำเนินประสานงานพยาบาลหลังคลอด และส่งต่อจิตแพทย์ เพื่อให้การดูแลช่วยเหลือทางด้านอารมณ์และจิตใจอย่างเหมาะสม

<u>6 สัปดาห์หลังคลอด</u> ผู้วิจัยติดต่อกลุ่มควบคุมผ่านทาง line official account "Parent พาเพลิน" เพื่อทำแบบประเมินความเครียดในการเลี้ยงดูบุตร และแบบประเมินสมรรถนะในการเลี้ยงดูบุตร (Post-test) ผ่าน Link microsoft forms

กลุ่มทดลอง

<u>24 ชั่วโมงหลังคลอด</u> ผู้วิจัยสัมภาษณ์ข้อมูลส่วนบุคคลและให้ทำแบบประเมินความเครียดในการเลี้ยงดูบุตร และแบบประเมินสมรรถนะในการเลี้ยงดูบุตร (Pre-test) ผ่าน Link microsoft forms

โปรแกรมส่งเสริมการเลี้ยงดูบุตรร่วมกับ Line official account "Parent พาเพลิน"

- <u>ระยะ 48 ชั่วโมงหลังคลอด</u> สร้างสัมพันธภาพ แนะนำตัวเพื่อสร้างสัมพันธภาพที่ดีกับมารดาวัยรุ่น จากนั้นผู้วิจัยส่งเสริมสัมพันธภาพระหว่างมารดา - ทารก ให้ความรู้และฝึกทักษะรายบุคคลเกี่ยวกับสื่อสัญญาณทารกและการตอบสนองต่อสื่อสัญญาณทารก การดูแลบุตร การเลี้ยงบุตรด้วยนมมารดา การอาบน้ำบุตร และการสังเกตอาการผิดปกติของทารก โดยใช้สื่อการสอนในรูปแบบของ infographics, e-book, วิดีทัศน์ ผ่าน line official account "Parent พาเพลิน" โดยจะต้อง add line official account เพื่อเข้าถึงเนื้อหา และสามารถใช้สำหรับทบทวนได้ ใช้เวลา 60 – 90 นาที
- <u>ระยะ 48 – 72 ชั่วโมงหลังคลอด</u> ทบทวนความรู้ ฝึกทักษะเพิ่มเติมเพื่อส่งเสริมความมั่นใจของมารดา หากมารดาวัยรุ่นสามารถปฏิบัติได้ดี กล่าวชมเชยและให้กำลังใจ แต่หากมารดาวัยรุ่นยังขาดความมั่นใจ ดูแลให้ความช่วยเหลือรายบุคคลตามปัญหาที่เกิดขึ้นในแต่ละบุคคล ใช้เวลา 60 นาที และเตรียมจำหน่ายออกจากโรงพยาบาล

<u>1 สัปดาห์หลังคลอด</u> ทำแบบประเมินภาวะซึมเศร้าหลังคลอด หากมีภาวะซึมเศร้าหลังคลอด ผู้วิจัยจะไม่คัดออกจากการวิจัย แต่ดำเนินประสานงานพยาบาลหลังคลอด และส่งต่อจิตแพทย์ เพื่อให้การดูแลช่วยเหลือทางด้านอารมณ์และจิตใจอย่างเหมาะสม

<u>สัปดาห์ที่ 1 – 5 หลังคลอด</u> ติดตามภายหลังการจำหน่าย ผ่าน line official account "Parent พาเพลิน" ทุกสัปดาห์ สัปดาห์ละ 1 ครั้ง โดยสอบถามปัญหาและให้คำปรึกษา

<u>6 สัปดาห์หลังคลอด</u> ผู้วิจัยติดต่อกลุ่มทดลองผ่านทาง line official account "Parent พาเพลิน" เพื่อทำแบบประเมินความเครียดในการเลี้ยงดูบุตร และแบบประเมินสมรรถนะในการเลี้ยงดูบุตร (Post-test) ผ่าน Link microsoft forms

รูปภาพ 3: ขั้นตอนการดำเนินการเก็บข้อมูล

INSTITUTION REVIEW BOARD
Queen Savang Vadhana Memorial Hospital
IRB No. ....01.../..2566..........
Date of Approval ....- 6 ก.พ. 2566....

12. การวิเคราะห์ข้อมูลและสถิติที่ใช้วิเคราะห์ (Data analysis and statistics)

ผู้วิจัยวิเคราะห์ข้อมูล โดยใช้โปรแกรมสำเร็จรูปทางสถิติ SPSS โดยกำหนดระดับนัยสำคัญที่ .05 และใช้สถิติในการวิเคราะห์ดังนี้

1. วิเคราะห์ข้อมูลส่วนบุคคล ใช้สถิติพรรณนา (descriptive statistics) โดยการแจกแจงความถี่ ร้อยละ ค่าเฉลี่ย และส่วนเบี่ยงเบนมาตรฐาน
2. ทดสอบความแตกต่างคุณลักษณะของกลุ่มตัวอย่างในด้านของจำนวนครั้งการตั้งครรภ์, ความต้องการการมีบุตร, สถานภาพการสมรส, ประสบการณ์การเลี้ยงดูทารก และบุคคลช่วยเหลือในการเลี้ยงดูบุตร โดยใช้สถิติไคสแควร์ (Chi-square test)
3. เปรียบเทียบค่าเฉลี่ยคะแนนความเครียดในการเลี้ยงดูบุตร และสมรรถนะในการเลี้ยงดูบุตร ก่อนและหลังการเข้าร่วมโปรแกรม โดยใช้สถิติทดสอบค่าทีแบบสองกลุ่มที่มีความสัมพันธ์กัน (Dependent T-test)
4. เปรียบเทียบค่าเฉลี่ยคะแนนความเครียดในการเลี้ยงดูบุตร และสมรรถนะในการเลี้ยงดูบุตร ของมารดาวัยรุ่นภายหลังได้รับโปรแกรม 6 สัปดาห์หลังคลอดระหว่างกลุ่มทดลองและกลุ่มควบคุม โดยใช้สถิติทดสอบค่าทีแบบสองกลุ่มที่ไม่มีความสัมพันธ์กัน (Independent T-test)

13. ข้อพิจารณาด้านจริยธรรม (Ethical consideration)

ผู้วิจัยขอความร่วมมือพยาบาลหอผู้ป่วยหลังคลอดสามัญในการคัดเลือกกลุ่มตัวอย่างที่มีคุณสมบัติตรงตามเกณฑ์ที่กำหนด โดยพยาบาลหอผู้ป่วยหลังคลอดสามัญขออนุญาตให้ผู้เข้าวิจัยเข้าพบ เมื่ออาสาสมัครอนุญาต ผู้วิจัยจึงเข้าพบอาสาสมัครเพื่อชี้แจงรายละเอียดของโครงการวิจัย โดยอธิบายวัตถุประสงค์ของการวิจัย ขั้นตอนของการเก็บข้อมูล ประโยชน์ที่ได้รับและความเสี่ยงที่อาจเกิดขึ้นจากการเข้าร่วมโครงการวิจัย และเปิดโอกาสให้อาสาสมัครได้ซักถามข้อสงสัย พร้อมทั้งตอบข้อสงสัยจากอาสาสมัคร โดยอาสาสมัครเป็นผู้ตัดสินใจที่จะเข้าร่วมการวิจัยด้วยตนเอง มีสิทธิปฏิเสธการเข้าร่วมการวิจัย มีสิทธิที่จะปฏิเสธการตอบคำถามในข้อที่ไม่ต้องการตอบ และถอนตัวจากการเข้าร่วมวิจัยได้ตลอดเวลาโดยไม่มีผลกระทบใด ๆ ต่อการบริการที่กลุ่มตัวอย่างจะได้รับ หากอาสาสมัครมีความประสงค์ที่จะเข้าร่วมโครงการวิจัย จึงให้ลงนามในใบยินยอมเข้าร่วมการวิจัย ในกรณีอาสาสมัครอายุน้อยกว่า 18 ปี ต้องได้รับความยินยอมจากผู้ปกครองหรือสามีก่อน

ข้อมูลที่อาจนำไปสู่การเปิดเผยตัวของอาสาสมัคร จะได้รับการปกปิดและจะไม่เปิดเผยแก่สาธารณชน ในกรณีที่ผลการวิจัยได้รับการตีพิมพ์ ชื่อและที่อยู่ของอาสาสมัครจะได้รับการปกปิดอยู่เสมอ โดยจะใช้เฉพาะรหัสประจำโครงการวิจัยของท่าน การนำเสนอผลการวิจัยเป็นไปในภาพรวม ไม่มีการชี้เฉพาะถึงตัวบุคคล และข้อมูลที่ได้จะเป็นประโยชน์ในทางวิชาการเท่านั้น

14. ประโยชน์ที่คาดว่าจะได้รับจากการวิจัย (Expected benefit)

ผลผลิตที่ได้จากการวิจัย

1. นวัตกรรม Line official account "Parent พาเพลิน" และดำเนินการจดลิขสิทธิ์

ประโยชน์ที่คาดว่าจะได้รับ

1. ด้านการวิจัย เพื่อเป็นแนวทางในการพัฒนาโปรแกรมการส่งเสริมการเลี้ยงดูบุตรของมารดาวัยรุ่น หรือพัฒนานวัตกรรมในรูปแบบ Line official account ในประเด็นอื่น ๆ เช่น มารดาวัยรุ่นที่มีภาวะแทรกซ้อน เพื่อให้เกิดองค์ความรู้ทางการพยาบาลต่อไป
2. ด้านการปฏิบัติการพยาบาล เพื่อเป็นแนวทางสำหรับบุคลากรทางสุขภาพในการพัฒนารูปแบบการดูแลมารดาวัยรุ่นเพื่อส่งเสริมให้มารดาวัยรุ่นมีสมรรถนะในการเลี้ยงดูบุตรที่เหมาะสม
3. ด้านการศึกษาพยาบาล เพื่อเป็นแนวทางในการบูรณาการกับการเรียนการสอนภาคปฏิบัติให้แก่นักศึกษาพยาบาลในการประยุกต์ใช้โปรแกรมการส่งเสริมการเลี้ยงดูบุตรในการดูแลมารดาวัยรุ่น

INSTITUTION REVIEW BOARD
Queen Savang Vadhana Memorial Hospital
IRB No. ....01....... /......2566..........
Date of Approval ....- 6 ก.พ. 2566..........

15. ความเสี่ยงที่จะเกิดขึ้นและความรับผิดชอบ (Risk and Investigator's responsibility)

การวิจัยครั้งนี้ไม่มีการปฏิบัติการใดๆ ที่เป็นความเสี่ยงที่ทราบหรือทำให้อาสาสมัครได้รับบาดเจ็บ การดำเนินโครงการวิจัยเป็นเพียงการให้ความรู้และการส่งเสริมสมรรถนะในการเลี้ยงดูบุตร และการตอบแบบสอบถาม ซึ่งอาจจะทำให้อาสาสมัครรู้สึกเหนื่อยล้า หรืออึดอัดไม่สบายใจ ในระหว่างเข้าร่วมโครงการวิจัย ถ้าหากอาสาสมัครไม่สะดวกหรือต้องการยกเลิกการเข้าร่วมโครงการวิจัยสามารถยุติได้ตลอดเวลา หรืออาจจะไม่ตอบแบบสอบถามใดก็ได้ที่รู้สึกว่าไม่ต้องการที่จะให้ข้อมูล ในกรณีที่ผู้วิจัยพบว่า อาสาสมัครมีภาวะแทรกซ้อนในระยะหลังคลอดภายหลังการจำหน่ายออกจากโรงพยาบาล หรือทำแบบประเมินภาวะซึมเศร้าหลังคลอดคะแนนเท่ากับ 11 คะแนนขึ้นไป หรือมีคะแนนในข้อสุดท้าย ซึ่งเป็นข้อคำถามเกี่ยวกับความคิดในการฆ่าตัวตาย แสดงถึงมีภาวะซึมเศร้าหลังคลอด ผู้วิจัยจะไม่คัดออกจากการวิจัย ผู้วิจัยจะดำเนินช่วยเหลือโดยการประสานงานกับพยาบาลประจำหอผู้ป่วยที่เกี่ยวข้อง เพื่อส่งต่อแพทย์ในการดูแลอย่างเหมาะสมต่อไป

หากพบอันตรายที่เกิดขึ้นจากการวิจัย อาสาสมัครจะได้รับการดูแลอย่างเหมาะสมทันที ในกรณีที่ท่านได้รับอันตรายใด ๆ หรือต้องการข้อมูลเพิ่มเติมที่เกี่ยวข้องกับโครงการวิจัย ท่านสามารถติดต่อกับผู้ทำวิจัยคือ อาจารย์สุนีย์ กลีบปาน และผู้ช่วยศาสตรจารย์ ดร. พรพิมล อาภาสสกุล ที่อยู่ สถาบันการพยาบาลศรีสวรินทิรา สภากาชาดไทย 1873 ถนนพระราม 4 แขวงปทุมวัน เขตปทุมวัน กรุงเทพมหานคร 10330 โทรศัพท์ 092-461-4926 ได้ตลอด 24 ชั่วโมง

16. ระยะเวลาที่ใช้ในการวิจัยตั้งแต่เริ่มต้นจนสิ้นสุดโครงการ (Timeline)

มกราคม 2565 - ธันวาคม 2566

17. สถานที่ทำวิจัย (Venue of the study)

หอผู้ป่วยสูติกรรมสามัญ โรงพยาบาลสมเด็จพระบรมราชเทวี ณ ศรีราชา สภากาชาดไทย

18. การบริหารงานวิจัยและตารางการปฏิบัติงาน (Research activities and timeline)

กิจกรรม	พ.ศ. 2565												พ.ศ. 2566											
	มค.	กพ.	มีค.	เมย.	พค.	มิย.	กค.	สค.	กย.	ตค.	พย.	ธค.	มค.	กพ.	มีค.	เมย.	พค.	มิย.	กค.	สค.	กย.	ตค.	พย.	ธค.
เสนอโครงการขอรับทุนอุดหนุนการวิจัยรอบ Concept	↔																							
ปรับแก้โครงการและพัฒนาเครื่องมือวิจัย		←	—	—	—	—	—	—	—	—	→													
เสนอโครงการขอรับการพิจารณาจริยธรรมการวิจัย												←	→											
ขออนุญาตเก็บรวบรวมข้อมูล														↔										
ดำเนินการเก็บรวบรวมข้อมูล															←	—	—	—	—	—	—	—	→	
วิเคราะห์ข้อมูลและอภิปรายผลการวิจัย																								↔
จัดทำรูปเล่มรายงานการวิจัยฉบับสมบูรณ์																								↔
ตีพิมพ์และเผยแพร่ผลงานวิจัย																								↔

INSTITUTION REVIEW BOARD
Queen Savang Vadhana Memorial Hospital
IRB No. ....01.... /...2566...
Date of Approval ....- 6 ก.พ. 2566....

19. งบประมาณค่าใช้จ่ายในการวิจัย (Budget)

หมวดค่าใช้จ่าย	จำนวน	หน่วยละ	งบประมาณ (บาท)
**1.หมวดค่าตอบแทน**			
- ค่าตอบแทนผู้วิจัย	2 คน	15,000	30,000
- ค่าตอบแทนผู้ทรงคุณวุฒิในการตรวจสอบคุณภาพของเครื่องมือที่ใช้ในการวิจัย	5 คน	1,000	5,000
**รวมหมวดค่าตอบแทน**			**35,000**
**2. หมวดค่าจ้าง**			
- ค่าจ้างผู้ช่วยวิจัย พยาบาลหลังคลอด (คัดเลือกกลุ่มตัวอย่าง, เก็บข้อมูล และประสานงาน) จำนวน 6 เดือน เดือนละ 2,000 บาท	1 คน	12,000	12,000
- ค่าจ้างในการพัฒนาสื่อ Graphic สำหรับ line official account			50,000
**รวมหมวดค่าจ้าง**			**62,000**
**3. หมวดค่าใช้สอย**			
- ค่าจ้างพิมพ์และค่าถ่ายเอกสาร			5,000
- ค่าจัดทำรูปเล่มรายงานฉบับสมบูรณ์			5,000
- ค่าสืบค้นข้อมูล			3,000
- ค่าวิเคราะห์ข้อมูล			5,000
- ค่าเดินทางในการเก็บข้อมูล	6 เที่ยว	4,800	4,800
**รวมหมวดค่าใช้สอย**			**22,800**
**4. หมวดวัสดุ**			
- ค่าวัสดุสำนักงาน			3,000
- ค่าวัสดุคอมพิวเตอร์			3,000
- ค่าชุดของขวัญทารกแรกเกิด สำหรับเป็นของที่ระลึกกลุ่มตัวอย่าง	60 ชุด	300	18,000
- ค่าจัดส่งของที่ระลึกทางไปรษณีย์	60 ชิ้น	50	3,000
**รวมหมวดวัสดุ**			**27,000**
**5. หมวดสาธารณูปโภค**			
- ค่าเครือข่ายอินเตอร์เน็ต			1,500
**รวมหมวดสาธารณูปโภค**			***1,500***
**รวมงบประมาณดำเนินการ**			***148,300***
**ค่าตอบแทนผู้ทรงคุณวุฒิในการพัฒนาการวิจัย (ร้อยละ 10)**	1 ท่าน		***14,830***
**รวมงบประมาณทั้งหมด**			***163,130***

20. เอกสารอ้างอิง (References)

กระทรวงดิจิทัลเพื่อเศรษฐกิจและสังคม. (2563). *รายงานผลการสำรวจพฤติกรรมผู้ใช้อินเตอร์เน็ตในประเทศไทย ปี 2563.* สำนักยุทธศาสตร์ สำนักพัฒนาธุรกรรมทางอิเล็กทรอนิกส์ กระทรวงดิจิทัลเพื่อเศรษฐกิจและสังคม.

ขนิษฐา เมฆกมล และอารีรัตน์ วิเชียรประภา. (2561). บทบาทพยาบาลในการดูแลมารดาวัยรุ่น. *วารสารวิจัยทางวิทยาศาสตร์สุขภาพ,* 12(2), 69 – 77.

จารีศรี กุลศิริปัญโญ. (2561). วัยรุ่นตั้งครรภ์:จุดเปลี่ยนที่ท้าทาย. ใน *การประชุมวิชาการและนำเสนอผลงานวิจัยระดับชาติ ครั้งที่ 11 เรื่อง "นวัตกรรมงานวิจัย สู่การสร้างสรรค์พัฒนาสังคมไทยอย่างยั่งยืน"* (น. 448-454). ปทุมธานี, มหาวิทยาลัยเวสเทิร์น.

วิสา การบุญ. (2560). *ปัจจัยที่ส่งผลต่อการรับรู้ข่าวสารผ่าน Line official account ของบุคลากรทางการแพทย์.* (สารนิพนธ์ปริญญาการจัดการมหาบัณฑิต วิทยาลัยการจัดการ). มหาวิทยาลัยมหิดล.

ศิริรัตน์ อินทรเกษม, พยอม สินธุศิริ, ดารุนนีย์ สวัสดิโชตติ์, ดัชนี สุวรรณคม, จรินทร โคตรพรม และวรนุช ไชยวาน. (2562). แนวทางในการส่งเสริมความสำเร็จในการดำรงบทบาทการเป็นมารดาวัยรุ่น:กรณีศึกษาจังหวัดนครพนม. *วารสารพยาบาลทหารบก,* 20(1), 128-137.

INSTITUTION REVIEW BOARD
Queen Savang Vadhana Memorial Hospital
IRB No. 01 /2566
Date of Approval - 6 ก.พ. 2566

สุนีย์ กลีบปาน, วรรณา พาหุวัฒนกร, ฉวีวรรณ อยู่สำราญ และวิมลนันท์ พุฒิวณิชพงศ์. (2562). ปัจจัยที่มีอิทธิพลต่อสมรรถนะในการเลี้ยงดูบุตรในมารดาหลังคลอดบุตรคนแรก. *วารสารพยาบาลทหารบก*, 20(2), 140-149.

สุนทรี ศรีโกไสย, พัชรี หมื่นชัย, ชฎาพร คำฟู, ศิริวรรณ ทวีวัฒนปรีชา และรัตนา สายพานิชย์. (2563). ความตรงและความเชื่อถือได้ของดัชนีชี้วัดความเครียดผู้ปกครองของเด็กอายุ 1 เดือน ถึง 12 ปี. *วารสารสุขภาพจิตแห่งประเทศไทย*, 28(1), 56-71.

สุริยา ยอดทอง, รุ่งฤดี อุสาหะ และอาภรณ์ ภู่พัทธยากร. (2563). ความเครียดและการเผชิญความเครียดของมารดาวัยรุ่นครรภ์แรกหลังคลอด. *วารสารการแพทย์โรงพยาบาลศรีสะเกษ สุรินทร์ บุรีรัมย์*, 35(2), 381 – 391.

สำนักอนามัยการเจริญพันธุ์ กระทรวงสาธารณสุข. (2560). *ยุทธศาสตร์การป้องกันและแก้ไขปัญหาการตั้งครรภ์ในวัยรุ่นระดับชาติ พ.ศ. 2560-2569*. นนทบุรี, สำนักอนามัยการเจริญพันธุ์ กรมอนามัย กระทรวงสาธารณสุข.

สำนักอนามัยการเจริญพันธุ์ กระทรวงสาธารณสุข. (2563). *รายงานประจำปี 2563 สำนักอนามัยการเจริญพันธุ์*. นนทบุรี, สำนักอนามัยการเจริญพันธุ์ กรมอนามัย กระทรวงสาธารณสุข.

Abidin, R. R. (1995). *Parenting stress index: Manual (3rd ed.)*. Odessa, FL: Psychological Assessment Resources.

Althabe, F., Moore, J. L., Gibbons, L., Berrueta, M., Goudar, S. S., Chomba, E., ... McClure, E. M. (2015). Adverse Maternal and Perinatal Outcomes in Adolescent Pregnancies: The Global Network's Maternal Newborn Health Registry Study. Reproductive health, 12(2), 1-9.

Angley, M., Divney, A., Magriple, U., & Kershaw, T. (2015). Social support, family functioning and parenting competence in adolescent parents. *Maternal and Child Health Journal*, 19(1), 67-73.

Barabach, L., Ludington-Hoe, S. M., Dowling, D. & Lotas, M. (2017). Role of baby-friendly hospital care in maternal role competence. *Nursing for Women's Health*, 21(2), 96-107.

Belsky, J. (1984). The determinants of parenting: A process model. *Child Development*, 55(1), 83–96.

Brown, S. G., Hudson, D. B., Grossman, C. C., Kupzyk, K. A., Yates, B. C., & Hanna, K. M. (2016). Social Support, Parenting Competence, and Parenting Satisfaction among Adolescent, African American, Mothers. *Western Journal of Nursing Research*, 1-18.

Corcoran, J. (2016). Teenage Pregnancy and Mental Health. *Societies Journal*, 6, 1-9.

Erfina, E., Widyawati, W., McKenna, L., Reisenhofer, S., & Ismail, D. (2019). Adolescent Mothers' Experiences of the Transition to Motherhood: An Integrative Review. *International Journal of Nursing Sciences*, 6(2), 221-228.

Kershaw, T., Murphy, A., Lewis, J., Divney, A., Albritton, T., Magriples, U., et al. (2014). Family and relationship influences on parenting behaviors of young parents. *Journal of Adolescent Health*, 54, 197-203.

Kroja, R., Latva, R., & Lehtonen, L. (2012). The effects of preterm birth on mother-infant interaction and attachment during the infant's first two years. *Acta Obstet Gynecol Scand*, 91, 164-173.

Maehara, K., Mori, E., Tsuchiya, M., Iwata, H., Sakajo, A., Ozawa, H., et al. (2016). Factors affecting maternal confidence among older and younger Japanese primiparae at one month post-partum. *Japan Journal of Nursing Science*. doi: 10.1111/jjns.12123

Mangeli, M., Rayyani, M., Cheraghi, M. A., & Tirgari, B. (2017). Exploring the Challenges of Adolescent Mothers from Their Life Experiences in the Transition to Motherhood: A Qualitative Study. *Journal of Family and Reproductive Health*, 11(3), 165-173.

Martin, J. A., Hamilton, B. E. & Osterman, M. J. K. (2021). Births in the United States, 2020. *NCHS Data Brief*, 418, 1-8.

Moudi, Z., Talebi, B. & Pour, M. S. (2017). Effect of a brief training program for primigravid adolescents on parenting self-efficacy and mother-infant bonding in the southeast of Iran. *Int J Adolesc Med Health*, 32(1).

Ngai, F. W., & Chan, S. W. C. (2012). Stress, Maternal Role Competence, and Satisfaction among Chinese women in the perinatal period. *Research in Nursing & Health*, 35, 30-39.

Ogawa, K., Matsushima, S., Urayama, K. Y., Kikuchi, N., Nakamura, N., Tanigaki, S., ...Morisaki, N. (2019). Association between adolescent pregnancy and adverse birth outcomes, a multicenter cross sectional Japanese study. *Scientific Reports*, 9, 1-8.

Osok, J., Kigamwa, P., Stoep, A. V., Huang, K. Y., & Kuma, M. (2018). Depression and psychosocial risk factors associated with pregnancy in Kenyan adolescents: A cross-sectional study in a community health center of Nairobi. *BMC Psychiatry*, 18(1), 1-10.

Ponomartchouk, D., & Bouchard, G. (2015). New mothers' sense of competence: predictors and outcomes. *Journal of Child and Family Studies*, 24, 1977–1986.

Shrooti, S., Mangala, S., Nirmala, P., Devkumari, S., Dharanidhar, B. (2016). Perceived maternal role competence among the mothers attending immunization clinics of Dharan, Nepal. *International Journal of Community Based Nursing and Midwifery*, 4(2), 100-106.

Shorey, S., Chan, S. W. C., Chong, Y. S., & He, H. G. (2015). Predictors of maternal parental self-efficacy among primiparas in the early postnatal period. *Western Journal of Nursing Research*, 37(12), 1604-1622.

United Nations. (2019). *progress towards the Sustainable Development Goal's Report of the Secretary-General*. New York, Economic and Social Council, United Nations.

Vance, A. J., & Brandon, D. H. (2017). Delineating among parenting confidence, parenting self-efficacy, and competence. *Advances in Nursing Science*, 40(4), 18-37.

21. ลงนามรับรองโครงร่างการวิจัย

ผู้วิจัยขอรับรองว่าจะปฏิบัติตามขั้นตอนและวิธีการที่เขียนไว้ในโครงร่างการวิจัยฉบับนี้อย่างเคร่งครัด

ลงชื่อผู้วิจัย.......สุนิ่ง กลิ่นปาน.......

หน่วยงาน สาขาการพยาบาลมารดา ทารกและการผดุงครรภ์

สถาบันการพยาบาลศรีสวรินทิรา สภากาชาดไทย

วันที่ 1 เดือนกุมภาพันธ์ พ.ศ. 2566

INSTITUTION REVIEW BOARD

[illegible]een Savang Vadhana Memorial Hospital

[illegible] No. ....01 / 2566........

Date of Approval ....- 6 ก.พ. 2566........
