# Supplementary material for: The effect of parental enhancing program with mobile application on parental stress and competence among Thai adolescent postpartum women: A quasi-experimental matched control design
Source: PLoS One. 2025 Oct 31;20(10):e0324318. doi: 10.1371/journal.pone.0324318 (PMC12578233; doi:10.1371/journal.pone.0324318)
Supplement: S2 File — Study protocol (English translation version). (PDF) [file pone.0324318.s004.pdf]

Research Protocol

1. Research title

The Effect of Parental Enhancing Program with Line Official Account "Parent Paplearn" on Parental Stress and Parental Competence Among Adolescent Postpartum Mothers

2. Investigators

2.1 Principal investigator

Miss Sunee Kleebpan

Nursing Lecturer, Maternal – Newborn Nursing and Midwifery, Srisavarindhira Thai Red Cross Institute of Nursing, 1873 Rama IV Road, Pathumwan, Bangkok, 10330 Thailand

2.2 Co-investigators

Dr. Pornpimol Apartsakun

Assistant Professor, Maternal – Newborn Nursing and Midwifery, Srisavarindhira Thai Red Cross Institute of Nursing, 1873 Rama IV Road, Pathumwan, Bangkok, 10330 Thailand

2.3 Co-investigators

Mrs. Pensiri Chaiyanusak

Registered Nurse, Obstetric ward, Queen Savang Vadhana Memorial Hospital, 290 Jermjompol Road, Sriracha, Chonburi, 20110 Thailand

2.4 Co-investigators

Dr. Ellen Kitson-Reynolds

Principle Teaching Fellow, School of Health Sciences, Building 67, University of Southampton, Highfield, Southampton, Hants SO17 1BJ, UK

Research consultant

Dr. Nittaya Sinsuksai

Assistant Professor, Obstetrics and Gynecology, Faculty of Nursing, Mahidol University, 2 Wang Lang Road, Siriraj, Bangkok Noi, Bangkok 10700

3. Background and rationale

Adolescent pregnancy remains a significant public health concern worldwide, including in Thailand. According to the United Nations Sustainable Development Goals (SDGs) progress report, the global adolescent birth rate showed a slight decline, from 45 per 1,000 live births in

2015 to 44 in 2019 (United Nations, 2019). In the United States, the birth rate among females aged 15–19 years decreased from 17.4 per 1,000 in 2018 to 16.7 in 2019 (Martin, Hamilton, & Osterman, 2021). In Thailand, data from 2015 to 2019 indicate a consistent decline in adolescent births among females aged 15–19 years, with rates of 44.8, 42.5, 39.6, 35.0, and 31.3 per 1,000, respectively (Thailand Ministry of Public Health, 2020). Despite this downward trend, the rate remains above the target set by the SDGs, which aim to reduce adolescent birth rates among individuals aged 10–19 years by 2030. Furthermore, Thailand's National Strategy on Adolescent Pregnancy Prevention and Resolution (2017–2026) has established a national goal to reduce the birth rate among females aged 15–19 years to no more than 25 per 1,000 by 2026 (Thailand Ministry of Public Health, 2017).

Adolescent pregnancy remains stubbornly high, falling short of global and national reduction goals and hampering both maternal-child health and wider social and economic progress. Young mothers must cope with rapid physical, psychological, and social changes while taking on the responsibilities of parenthood, a transition often clouded by fear and anxiety about raising a child (Mangeli et al., 2017). Because they are still developing themselves, they may lack the knowledge, experience, and emotional maturity needed for confident caregiving, especially in the early postpartum period (Ngai & Chan, 2012). These challenges can undermine their sense of parenting efficacy and limit the quality of care they can provide.

Parental stress is the tension caregivers feel when child-rearing demands seem to outstrip their available resources. For adolescent mothers, who are still navigating their own development, the early postpartum period is especially sensitive. During these weeks, they must learn basic caregiving skills, bond with their infants, and decipher newborn cues. If they believe they are falling short of their own or others' expectations, anxiety can hinder their adjustment to the maternal role. To ease this stress, young mothers often turn to trusted confidants, seek guidance while still in the hospital, consult more experienced peers, or look for information online (Yodthong, Usaha, & Poopatayakon, 2020).

Parental competence refers to a mother's positive attitudes, practical knowledge, and caregiving skills, qualities that allow her to handle child-rearing tasks well and find satisfaction in motherhood. Many adolescent mothers struggle to develop this competence, however. The added responsibilities of parenting collide with their own ongoing physical and emotional changes, often leading to stress, anxiety, ineffective caregiving, and role conflict (Mangeli et al., 2017). Together, these pressures can undermine their confidence and caregiving ability, highlighting the need for age-appropriate, targeted interventions that build their parenting skills.

Effective support for adolescent mothers requires nurses to cultivate a positive attitude and maintain open communication, thereby facilitating an accurate understanding of each mother's needs. Such rapport can alleviate maternal anxiety and foster adaptation to the maternal role (Erfina et al., 2019; Intrakasem et al., 2019). Care should emphasise role adjustment through education, hands-on skill-building in infant care and breastfeeding, and structured activities that strengthen the mother–infant bond, all of which promote maternal confidence. Because adolescents are frequent users of digital technology, integrating social media and other online platforms into postpartum care represents an essential component of contemporary service models (Mekkamon & Wichienprapha, 2018). Current postpartum practice, however, often falls short of these ideals. Routine hospital discharge within 2–3 days limits opportunities for comprehensive and continuous skill development, and most institutions provide no structured follow-up or communication system after discharge, relying instead on printed manuals for self-study. This discontinuity may heighten anxiety and undermine caregiving effectiveness once mothers return home.

A review of the literature, corroborated by the researcher's clinical experience, indicates that postpartum care for adolescent mothers remains largely indistinguishable from that offered to adult mothers. Instruction is typically delivered through lectures, demonstrations, and brief skills training sessions. Yet adolescent mothers are seldom provided with accessible, engaging resources for later review: most information is still distributed as manuals, pamphlets, or compact discs, formats that neither match adolescents' media preferences nor sustain their attention. Opportunities for convenient follow-up consultation are likewise scarce. Accordingly, this study seeks to design and evaluate a technology-enhanced care model tailored to the specific needs of adolescent mothers.

The LINE application is currently one of the most widely used communication platforms in Thailand. A 2020 survey reported approximately 47 million LINE users nationwide, with members of Generation Z spending an average of 12 hours 8 minutes online each day (Thailand Electronic Transactions Development Agency, 2020). Owing to its popularity and accessibility, LINE offers a promising channel for engaging adolescents. The LINE Official Account enables large-scale dissemination of text, images, videos, and announcements, while also permitting followers to send private messages for confidential consultation; these interactions are invisible to other users and incur no costs beyond routine internet connectivity. Consequently, leveraging a LINE OA for health-education delivery could strengthen communication and furnish adolescent mothers with a convenient, private avenue for obtaining advice and support from healthcare professionals.

The research team developed a Parental Enhancing Program with Line Official Account " Parent Paplearn". The platform delivers evidence-based content on infant cues and appropriate caregiver responses, newborn care, breastfeeding, infant bathing, and the early recognition of neonatal abnormalities. Materials are presented in multiple formats, infographics, e-books, and short videos, allowing adolescent mothers to review information and seek consultation at their convenience. By combining on-demand educational resources with a confidential communication channel, the programmed is designed to strengthen parenting competence and mitigate parenting stress among adolescent mothers. Findings from the evaluation of this intervention are expected to inform refinements to postpartum-care systems and to advance support for adolescent mothers in fulfilling their caregiving responsibilities effectively.

4. Review literature

The researcher reviewed relevant literature and studies as follows:

1. **Adolescent Pregnancy**

Adolescent pregnancy refers to pregnancy in females aged 10–19 years, measured at the time of childbirth. It can be further categorized into adolescent pregnancy (ages 15–19) and younger adolescent pregnancy (ages 10–14).

Impacts of Adolescent Pregnancy

1. **Physical** Adolescents' bodies are still developing, which may increase health risks compared to adults. Most adolescent pregnancies are unplanned, potentially leading to inadequate prenatal care and behaviors, resulting in complications such as anemia, insufficient weight gain during pregnancy, pregnancy-induced hypertension, difficult labor, preterm birth, postpartum hemorrhage, low birth weight, miscarriage, or neonatal death (Althabe et al., 2015; Ogawa et al., 2019).

2. **Psychological** Mental health issues are common among pregnant adolescents. They often experience shame, guilt over unintended pregnancy, and social or familial rejection, which can cause anxiety, stress, and may develop into depression (Corcoran, 2016; Osok et al., 2018).

3. **Family** Families of pregnant adolescents often face stress, anger, and disappointment. Social stigma associated with adolescent pregnancy adds further burden, increasing the caregiving responsibilities for both the adolescent mother and her child (Jareesri, 2018).

4. **Social and Economic** A significant social issue related to adolescent pregnancy is illegal abortion, which can lead to severe health complications such as hemorrhage and

infection. Additionally, inadequate child-rearing or abandonment may occur, resulting in societal challenges and lower quality future workforce. The government incurs costs for healthcare and social welfare related to adolescent pregnancy (Jareesri, 2018).

2. Concept of the Maternal Role

2.1 Theory of Maternal Role

Rubin (1975) developed the Theory of Maternal Identity, which involves the following processes:

Replication: Women mimic or model their maternal behavior after their own mothers or other maternal figures.

Fantasy: Women imagine how they will perform their maternal role and how they will care for their child in the future.

Dedifferentiation: Women learn the maternal role by seeking role models among close individuals, then choose a model that fits them or reject roles they find unsuitable.

Identity Formation: Women establish a maternal identity by adopting a role that suits them, accepting their new role, expressing satisfaction, and effectively fulfilling maternal responsibilities.

In 1985, Mercer expanded on Rubin's concept, describing maternal role development as a continuous and dynamic process from pregnancy through postpartum, consisting of four stages (Mercer, 2004):

1. **Commitment, Attachment, and Preparation** This stage occurs during pregnancy, where women prepare for motherhood by caring for their health, seeking information on pregnancy care, developing an attachment to the fetus, and learning about the maternal role.
2. **Acquaintance, Learning, and Physical Restoration** Starting from childbirth until six weeks postpartum, women begin to enact the maternal role according to advice and expectations from others.
3. **Moving Toward a New Normal** From two weeks to four months postpartum, mothers increasingly express their maternal role in a way that reflects their own characteristics, based on experience and learning.
4. **Achievement of Maternal Identity** After four months postpartum, mothers gain confidence, competence in child-rearing, and satisfaction in fulfilling their maternal role.

Successful maternal role attainment can be evaluated by three components: 1) attachment to child, 2) confidence/ competence in role, and 3) gratification/ satisfaction.

2.2 Parental stress

Stress is a physiological and psychological response triggered by threatening stimuli (stressors), resulting in discomfort, anxiety, confusion, and imbalance.

Parental stress refers to the emotional reactions arising from various challenges encountered in child-rearing. Parenting is a new role that involves learning how to care for the child, building a parent-child relationship, and understanding the child’s behavior. If adolescent mothers are unable to meet their own or others’ expectations in caregiving, they may experience distress and anxiety. This can negatively affect their adjustment to the maternal role (Yodthong, Usaha, & Poopatayakon, 2020).

Abidin (1995) proposed that when parents are unable to effectively manage child-rearing tasks or respond appropriately to their child's needs in accordance with personal and societal expectations, they may experience a sense of failure in fulfilling their parental role, referred to as dysfunctional parenting. Based on this perspective, Abidin developed the Theoretical Model for the Parenting Stress Index (PSI), which explains the relationship between parental stress, parenting behavior, and child outcomes. The model comprises three core components:

1) Parental Distress: Refers to the emotional discomfort and psychological burden a parent experiences in fulfilling the caregiving role. It may stem from personal limitations, lack of support, or role dissatisfaction.

2) Parent–Child Dysfunctional Interaction: Represents the parent's perception that their child does not meet expectations and that interactions between the parent and child fail to generate a sense of satisfaction in the parental role. This often results in negative emotional experiences.

3) Difficult Child: Refers to the parent's perception of the child as temperamentally difficult or behaviorally challenging, making caregiving feel overwhelming and difficult to manage.

These components interact and contribute to overall parenting stress (Figure 1).

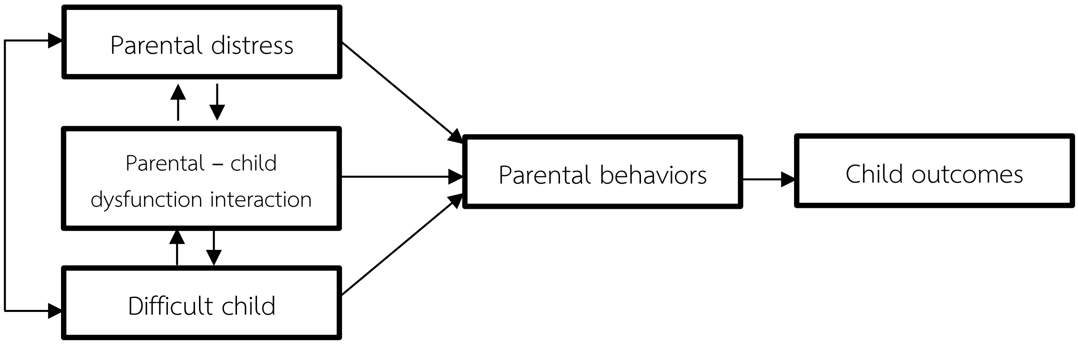

Figure 1 The conceptual framework diagram of Parenting Stress Index

2.3 Parental competence

Parental competence refers to parents' self-assessment of their ability to fulfill child-rearing responsibilities in accordance with personal or societal expectations. For instance, a mother may evaluate whether she is caring for her child effectively based on these standards (Vance & Brandon, 2017). Perceived parenting ability is closely associated with actual parental competence and reflects the overall quality of child-rearing.

Assessment of Parental Competence

Parental competence is assessed using the Parenting Sense of Competence Scale (PSOC), developed by Gibaud-Wallston and Wandersman (1978). The scale comprises two dimensions: 1) Efficacy: This dimension reflects the parents' perception of their ability to fulfill parenting responsibilities and solve problems related to child-rearing. 2) Satisfaction: This emotional dimension captures feelings such as anxiety, pride, and a sense of accomplishment in parenting.

3. Factors Influencing competent parental functioning

Belsky's "A Process Model of Competent Parental Functioning" (Belsky, 1984). to elucidate the various factors influencing parental roles in childcare. This model identifies three domains, including 1) 'Personal psychological resources of parents' domain encompasses individual characteristics. It has been found to exert the most significant influence on parental functioning. 2) 'Contextual sources of stress and support', which can either facilitate or hinder effective parenting, and 3) 'Characteristics of the Child', which may also influence parenting dynamics but generally have a lesser impact compared to the other two domains.

1. Personal psychological resources of parents

1.1 **Age** reflects developmental stages, such as adolescence or adulthood, which indicate an individual's readiness in physical, psychological, and social aspects. Adult mothers

tend to have better cognitive and problem-solving skills, which enhance their parenting competence (Shrooti et al., 2016).

1.2 **Marital Status** Single mothers bear the full responsibility of child-rearing, which may lead to increased stress and psychological challenges.

1.3 **Economic Status** Mothers with sufficient income tend to feel financially secure, enhancing their perceived parenting competence. In contrast, financial hardship can increase stress and lower parenting efficacy (Shrooti et al., 2016).

1.4 **Educational Level** Higher education improves information processing, problem-solving, and adaptability, contributing to more effective parenting (Shrooti et al., 2016).

1.5 **Parity** Previous childbearing experiences enhance maternal skills. Successful prior parenting experiences support more competent current parenting (Barabach et al., 2017).

1.6 **Physical Health** Chronic illness, complications, or medical conditions may require additional self-care, potentially reducing a mother's capacity to care for her child (Maehara et al., 2016).

1.7 **Pregnancy Readiness** Mothers who are prepared for pregnancy are more likely to engage in prenatal care and effective parenting (Shrooti et al., 2016).

1.8 **Self-Esteem** High self-esteem supports effective coping and adaptation, leading to improved parenting performance (Shrooti et al., 2016).

1.9 **Mental Health** Stress, anxiety, or depression may negatively affect parenting effectiveness (Angley et al., 2015; Shorey et al., 2015).

1.10 **Self-Efficacy** High maternal self-efficacy enhances confidence in parenting abilities, contributing to successful child-rearing practices.

2. **Contextual sources of stress and support**

2.1 **Social Support** enhances maternal parenting competence through the provision of resources, information, and emotional encouragement in child-rearing (Brown et al., 2016).

2.2 **Marital Relationship** A positive and caring relationship with a spouse fosters maternal support and reinforces parenting abilities (Kershaw, 2014).

3. Characteristics of the Child

3.1 **Child's Health Status** Infants with complications such as low birth weight, prematurity, or congenital disabilities may cause maternal stress, which can negatively affect parenting competence (Korja, Latva, & Lehtonen, 2012).

3.2 **Child's Temperament** Children who are difficult to soothe or prone to frequent crying may be perceived by mothers as challenging to manage, which can diminish maternal perceptions of parenting effectiveness and competence (Ponomartchouk & Bouchard, 2015).

4. Line official account in Health Promotion

The LINE Official Account is a feature of the LINE application designed for business or organizational use, allowing for the broader dissemination of information. It supports various multimedia formats, including text, images, posters, and videos, for convenient and effective communication with followers. Additionally, it enables two-way communication, allowing followers to send private messages for inquiries or consultations without other users seeing the exchange.

A key advantage of the LINE application is its ability to form group chats, were members, typically sharing similar experiences or characteristics, can communicate efficiently. A single message sent within the group reaches all members, enhancing group interaction. The application supports diverse file types (e.g., text, voice, images, videos, stickers, posters, and documents), enabling communication to be tailored to specific target audiences. Moreover, LINE reduces communication costs by allowing free voice calls through internet packages, eliminating the need for traditional phone charges (Kanabun, 2017)

Thailand, like the rest of the world, has entered the Digital 4.0 era, marked by the integration of intelligent technologies into various sectors, including healthcare. This transition has led to the rise of digital health platforms such as mobile applications, websites, electronic messaging, wearable devices, and health monitoring sensors, which aim to enhance the efficiency of healthcare services.

Among these tools, the LINE application has emerged as an effective communication platform, particularly well-suited to reaching adolescents. The LINE Official Account, designed for broad dissemination of information, allows for the easy sharing of messages, images, videos, and announcements with followers. It also enables private two-way communication, where followers can consult or inquire without their messages being visible to others.

As a free, internet-based communication channel, the LINE Official Account offers a private, accessible, and cost-effective means of interaction. Utilizing this platform to disseminate health information can improve communication between adolescent mothers and healthcare providers, offering a convenient and timely channel for consultation and support.

5. Objectives

1. Developed a Line Official Account “Parent Paplearn”.
2. To compare parenting stress among postpartum adolescent mothers who participated in the parental enhancing program with the mobile application through ‘Line Official Account™ Parent Paplearn’ and those receiving standard postpartum care.
3. To compare parenting competence among postpartum adolescent mothers who participated in the parental enhancing program with the mobile application through ‘Line Official Account™ Parent Paplearn’ and those receiving standard postpartum care.

6. Research framework

This study applies Belsky's “A Process Model of Competent Parental Functioning” (Belsky, 1984). to elucidate the various factors influencing parental roles in childcare. This model identifies three domains including 1) ‘Personal psychological resources of parents’ domain encompasses individual characteristics. It has been found to exert the most significant influence on parental functioning. 2) ‘Contextual sources of stress and support’, which can either facilitate or hinder effective parenting, and 3) ‘Characteristics of the Child’, which may also influence parenting dynamics but generally have a lesser impact compared to the other two domains.

The researchers utilized the parental competence framework to design the parental enhancing program with mobile application through ‘Line Official Account™ Parent Paplearn’. This program specifically targets contextual sources of stress and support, positioning nurses as vital resources to assist adolescent mothers in effectively caring for their children. In Thailand, nurses play a pivotal role in providing care through the nursing process, providing clients with appropriate education, and offering counselling. The program emphasizes the provision of knowledge and training in essential childcare skills, thereby enabling these mothers to perform their parenting roles with increased competence. Furthermore, it offers continuous support via mobile application through the ‘Line Official Account™ platform, facilitating timely responses to questions and concerns. This ongoing support not only alleviates parenting-related stress but also enhances the mothers' overall competence in childcare (Figure 2).

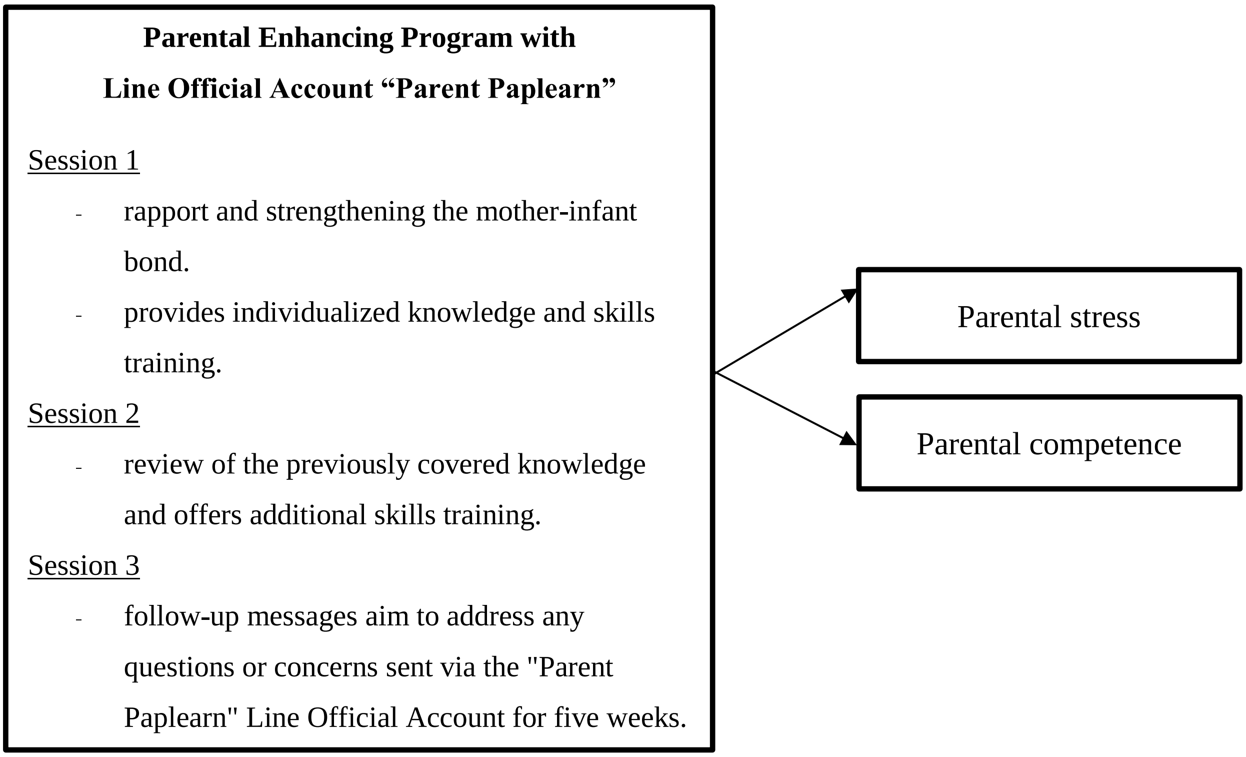

Figure 2. Research framework.

7. Research hypothesis

1. Participants in the intervention group experienced significantly lower levels of parental stress compared to those in the control group.
2. Participants in the intervention group experienced significantly higher scores in parental competence compared to those in the control group.

8. Keywords

Parental Program, Line Application, Parental Stress, Parental Competence, Adolescent Mothers

9. Research Design

This study employed a quasi-experimental control group pre-post-test design

10. Research methodology

10.1 Population

Postpartum women aged 15 to 19

10.2 Sample

The study sample consisted of postpartum women aged 15 to 19 who were admitted to the general obstetrics ward, Queen Savang Vadhana Memorial Hospital, Thai Red Cross Society, Thailand.

10.3 Sample size estimation

The sample size for this study was determined using G*Power (version 3.1.9.4), the effect size indicated a large effect (Cohen's guidelines), a power of test equal to 0.80, and a significance level set at 0.05. The analysis indicated that a sample size of 26 participants per group. To account for dropouts of 10 percent, the final sample was 30 participants per group. Consequently, the total sample size was established at 60 participants.

10.4 **Inclusion criteria**

1. Full-term deliveries (37-42 weeks)
2. Postpartum women aged 15 to 19
3. Mother without postpartum complications such as postpartum hemorrhage, preeclampsia with severe features, etc.
4. Infants exhibited no severe complications or congenital anomalies necessitating NICU admission or separation from the mother.
5. Mother intended to childcare for six-week
6. Participants could read, write, and communicate in Thai.
7. Demonstrated the ability to communicate via mobile using the LINE application.

Exclusion criteria

1. The participant did not personally care for the infant for the entire six-week postpartum period.
2. The participant was unable to complete all phases of the study.
3. The participant chose to withdraw from the research.

10.5 Informed consent process

The researcher explained the study objectives, procedures, and participants' rights. Informed consent forms were then obtained from the participants, with parental or spousal consent required for those under 18.

10.6 Instrument

10.6.1 Instruments for implementation

1. **The parental enhancing program**, developed by the researcher, is based on Belsky's "A Process Model of Competent Parental Functioning". The program consists of the following activities:

- Session 1 (24-48 hours postpartum): Establishes rapport and strengthens the mother-infant bond. The researcher provides personalized knowledge and skills training on recognizing infant cues and responsiveness, general infant care, breastfeeding techniques, bathing procedures, and identifying abnormal symptoms in infants, lasting about 60 to 90

minutes. Teaching materials, such as infographics, e-books, and instructional videos, are disseminated through the 'Line Official Account™ Parent Paplearn' as a mobile application.

- Session 2 (48-72 hours postpartum): Review the previously covered knowledge and offer additional skills training aimed at enhancing the mother's confidence. Individualized support is provided to address her specific concerns, lasting about 60 minutes.
- Session 3 (Post-discharge follow-up): Following discharge, the program includes weekly follow-up messages sent via mobile application through the 'Line Official Account™ Parent Paplearn' for five weeks to address any questions or concerns related to challenges faced by the mother or her infant.

2. **The Line Official Account™ Parent Paplearn** is a mobile application that facilitates the exchange of text messages, images, and videos on a broad scale. This platform also provides a private channel for adolescent postpartum women, enabling them to send messages for consultations or discussions regarding their parenting concerns. The platform is an online tool designed to enable users to create accounts, customize application designs, manage content, and perform system administration functions. The content available on 'Line Official Account™ Parent Paplearn' encompasses essential topics such as recognizing infant cues and responsiveness, general infant care, breastfeeding practices, bathing techniques, and identifying abnormal symptoms in infants. This information is delivered through engaging formats, including infographics, e-books, and videos, designed to enhance understanding and retention among adolescent mothers. The researchers developed the application interface, developed content, and primarily managed the system to address questions from adolescent postpartum women. Participants can review the knowledge as needed, without any limitation on the number of times.

10.6.2 Questionnaire or Assessment form

1. **The demographic characteristics questionnaire** collects data on the mother's age, marital status, education level, occupation, household income (THB), sufficiency of income, family structure, desire for children, plan for future education, childcare support, experience in childcare, number of pregnancies, type of delivery, and the infant's APGAR score.
2. **The postpartum problem and advice record** was developed by the researcher to systematically document follow-up interactions via mobile application through the 'Line Official Account™ Parent Paplearn' after hospital discharge, recording the date, problem, and advice provided.
3. **The Edinburgh Postnatal Depression Scale (EPDS)** was translated into Thai by Vacharaporn et al (2003). This screening tool consists of 10 items. For example, I have been

able to laugh and see the funny side of things, I have looked forward with enjoyment to things, etc. The total scores range from 0 to 30 points. A cut-off score of 11 or higher indicates potential depression. The EPDS serves as an important instrument in assessing the mental health needs of this population, facilitating timely interventions to promote overall well-being.

4. **The Parenting Stress Index Fourth Edition Short Form (PSI-4-SF)** utilized in this study employed the Thai version developed by Srikosa et al (2020). Originally 36 items, it was reduced to 15 to align with the context of the research, specifically focusing on infants aged six weeks postpartum. It measures three domains including 1) Parental distress, 2) Parent-child dysfunctional interaction, and 3) Difficult child, utilizing a five-point Likert scale. Scores range from 15 to 75 points, where lower scores indicate lower levels of parenting stress, while higher scores reflect greater parenting stress.

5. **The Parenting Sense of Competence Scale (PSOC)** was translated into Thai by Kleebpan et al (2019). This scale consists of 16 items measuring parenting efficacy and satisfaction. For example, the problems of taking care of a child are easy to solve once you know how your actions affect your child, an understanding you have acquired, etc. Scores ranged from 16 to 96 points. In this context, lower scores indicate lower levels of parenting competence, while higher scores reflect greater competence in parenting.

Instrument Quality Assessment

1. Validity

The research instruments were reviewed by three experts: two nursing professors specializing in maternal, infant, and obstetric nursing, and one nurse specialist in postpartum care. They evaluated the instruments for content accuracy, appropriate language, use of illustrations, alignment with the study objectives, and content comprehensiveness. The CVI scores for each instrument were as follows:

Line Official Account " Parent Paplearn": CVI = 1.00

The demographic characteristics questionnaire: CVI = 0.93

The postpartum problem and advice record: CVI = 1.00

The Edinburgh Postnatal Depression Scale (EPDS): CVI = 0.70

The Parenting Stress Index Fourth Edition Short Form (PSI-4-SF): CVI = 0.94

The Parenting Sense of Competence Scale (PSOC): CVI = 0.81

2. Reliability

The researcher tested the Line Official Account “Parent Paplearn”, the Parenting Stress Index Fourth Edition Short Form (PSI-4-SF), and the Parenting Sense of Competence Scale (PSOC) with 30 postpartum adolescent mothers whose characteristics closely matched the study

sample. This testing was conducted at Queen Savang Vadhana Memorial Hospital, Thai Red Cross Society, Thailand, following approval from the Ethics Committee. Reliability was then determined using Cronbach's alpha coefficient.

11. Data Collection

11.1 Participant Recruitment

The researcher collaborated with nurses to identify eligible participants who met inclusion criteria and obtained permission to meet them after 24 hours postpartum. During these meetings, the researcher explained the study objectives, procedures, and participants' rights. Informed consent forms were then obtained from the participants.

11.2Data Collection

1. The research proposal was submitted to the Ethics Committee of Queen Savang Vadhana Memorial Hospital, Thai Red Cross Society, to obtain permission for data collection.
2. Upon approval, the researcher submitted a formal request to the hospital director, which was forwarded to the Nursing Department and relevant ward supervisors to secure permission for data collection.
3. After receiving permission, the researcher introduced themselves to the ward supervisors, explained the study details, and requested cooperation in data collection.
4. The researcher collaborated with nurses to identify eligible participants who met inclusion criteria and obtained permission to meet them after 24 hours postpartum. During these meetings, the researcher explained the study objectives, procedures, and participants' rights. The nurses obtained consent from potential participants to allow the researcher to meet them. The sampling procedure for each group (Figure 3).

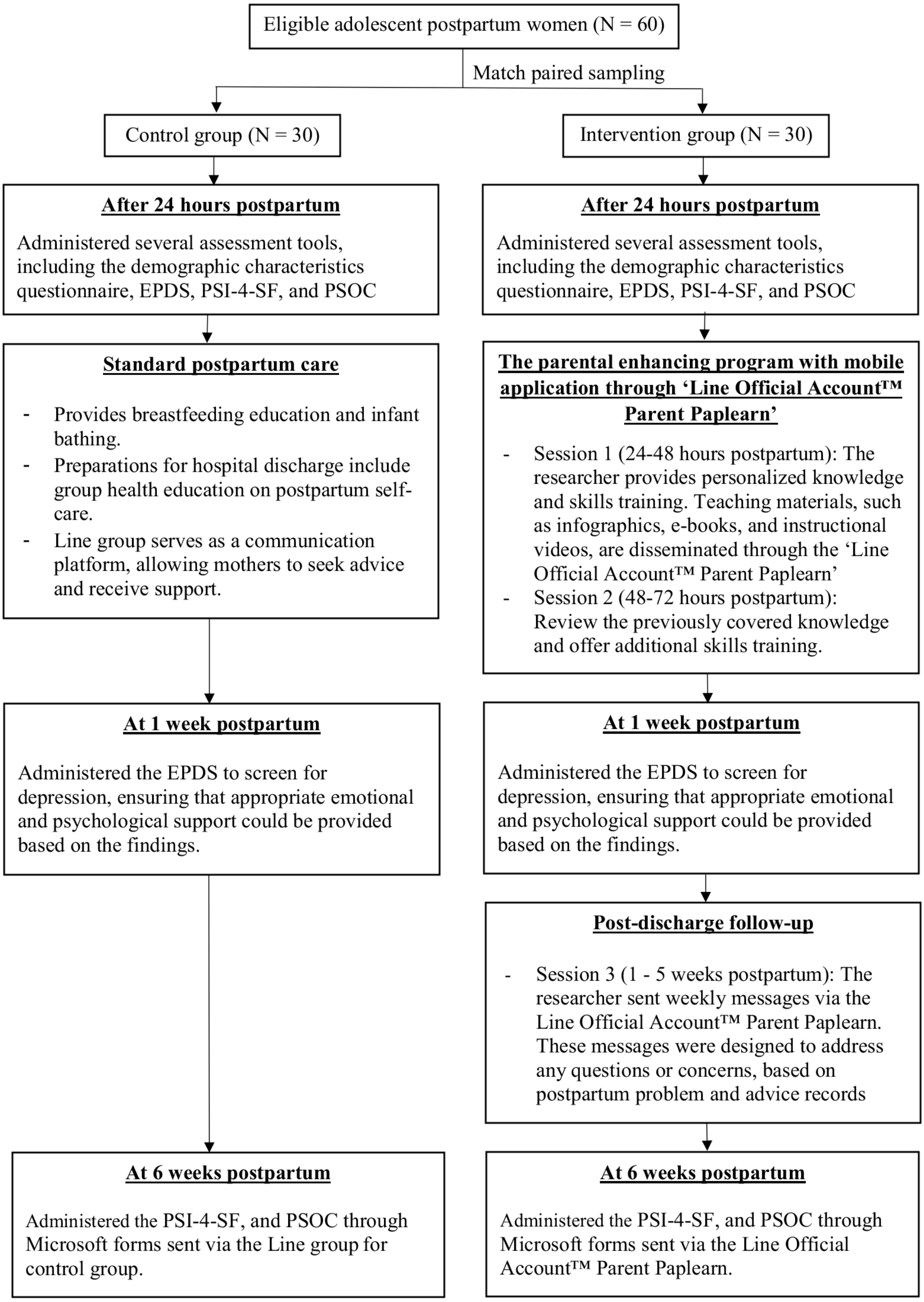

Figure 3. Flow diagram of study.

12. Data analysis and statistics

IBM SPSS Statistics was used for data analysis, statistical significance was set at $p < .05$.

1. Descriptive statistics were used to characterize the participant demographic and obstetric characteristics.

2. The comparison of differences between the characteristics of the intervention group and the control group was conducted using independent t-test, Chi-square test, and Fisher's exact test, based on type of the variables and assumptions of statistical.

3. The dependent t-test was used to determine significant differences in parental stress and competence scores before and after the intervention within each group.

4. The independent t-test was used to determine significant differences in parental stress and competence scores between the intervention group and the control group

13. Ethical consideration

The researcher collaborated with nurses to identify eligible participants who met inclusion criteria and obtained permission to meet them after 24 hours postpartum. During these meetings, the researcher explained the study objectives, procedures, and participants' rights. Participants were informed about the study, assured of confidentiality, and provided written consent, with parental or spousal consent required for those under 18. All potentially identifying information will remain confidential and will not be disclosed publicly. Should the findings be published, only project codes—rather than names or addresses—will be used. Results will be presented in aggregate, with no reference to individual participants, and the data will be employed exclusively for scholarly purposes.

14. Expected benefit

Research Output

1. The project resulted in the development of the LINE Official Account "Parent Paplearn".

Expected Benefits

1. **Research:** The innovation furnishes a framework for designing parenting support programs for adolescent mothers and for developing additional LINE based modules, such as those tailored to mothers experiencing obstetric complications, thereby extending the body of nursing knowledge.

2. **Clinical Practice:** It offers healthcare professionals a model for improving the care of adolescent mothers and for enhancing their parenting competence.

3. **Nursing Education:** It provides guidance for incorporating the parenting support program into clinical teaching, thereby enriching nursing students' practical training.

15. Risk and Investigator's responsibility

This study does not involve any procedures that present known risks or potential harm to participants. Its primary objective is to provide education and promote parenting competence, supplemented by the completion of questionnaires. Participants may experience fatigue or emotional discomfort during the process. Participation is entirely voluntary; individuals may withdraw from the study at any time or decline to answer any questions without penalty.

In cases where a participant exhibits postpartum complications following hospital discharge, scoring 11 or higher on the EPDS or indicating suicidal thoughts was not excluded. Instead, the researcher provided initial support by actively listening to their concerns and referred them to a nurse who facilitated further consultation with a psychiatrist, ensuring that they received appropriate emotional and mental health care.

If any harm results from participation in the study, appropriate care will be provided immediately. For any injury or to obtain further information regarding the research project, participants may contact the researchers, Miss Sunee Kleebpan and Assistant Professor Dr. Pornpimol Apartsakun, at Srisavarindhira Thai Red Cross Institute of Nursing, 1873 Rama IV Road, Pathumwan, Bangkok, 10330. They can be reached at 092-461-4926, available 24 hours.

16. Timeline

January 2022 – December 2023

17. Venue of the study

The general obstetrics ward, Queen Savang Vadhana Memorial Hospital, Thai Red Cross Society, Thailand.

18. Research activities and timeline

activities	2022												2023											
	Jan	Feb	Mar	Apr	May	Jun	Jul	Aug	Sep	Oct	Nov	Dec	Jan	Feb	Mar	Apr	May	Jun	Jul	Aug	Sep	Oct	Nov	Dec
Proposal Submission for Research Grant	↔																							
Project Revision and Research Instrument Development		↔	↔	↔	↔	↔	↔	↔	↔	↔	↔													
Ethics Approval Submission												↔	↔											
Permission for Data Collection														↔										
Data Collection															↔	↔	↔	↔	↔	↔	↔	↔	↔	
Data Analysis and Discussion																								↔
Final Report Preparation																								↔
Publication																								↔

19. Budget

Category	Quantity	Unit Cost (THB)	Total (THB)
**1. Personnel Compensation**			
Researcher	2 persons	15,000	30,000
Expert reviewers (instrument validation)	5 persons	1,000	5,000
**Subtotal**			**35,000**
**2. Wages**			
Research assistant (postpartum nurse: sampling, data collection, coordination)	1 person (6 months)	12,000	12,000
Graphic media development for LINE official account			50,000
**Subtotal**			**62,000**
**3. Operating Expenses**			
Printing and copying			5,000
Final report production			5,000
Data search fees			3,000
Data analysis			5,000
Fare for data collection	6 trips	4,800	4,800
**Subtotal**			**22,800**
**4. Materials**			
Office supplies			3,000
Computer accessories			3,000
Gift sets for participants	60 sets	300	18,000
Postage for gifts	60 packages	50	3,000
**Subtotal**			**27,000**
**5. Utilities**			
Internet service			1,500
**Subtotal**			***1,500***
**Total Research Budget**			***148,300***
**Honorarium for research consultant (10%)**	1 person		***14,830***
**Total Research Budget**			***163,130***

20. References

Thailand Electronic Transactions Development Agency. (2020). *Thailand Internet User Behavior 2020.* https://www.etda.or.th/th/Our-Service/statistics-and-information.aspx

Mekkamol, K., & Wichainprapha, A. (2018). The roles of nurses in teenage mother's caring [Thai]. *Journal of Health Science Research.* 12(2), 69–77.

Intharakasem, S., Sinthusiri, P., Sawaschote, D., Suwanakhom, D., Koteprom, J., & Chaiwan, W. (2019). Promoting of maternal role attainment achievement in adolescent mothers: A case study in Nakhon Phanom province [Thai]. *Journal of Royal Thai Army Nurses.* 20(1), 128–137.

Kleebpan, S., Phahuwatanakorn, W., Yusamran, C., & Putdivarnichapong, W. (2019). Factors influencing parental competence in first-time postpartum mothers [Thai]. *Journal of Royal Thai Army Nurses*. 20(2), 140–149.

Srikosai, S., Moanchai, P., Kamfou, C., Taweewattanaprecha, S., & Saipanish, R. (2020). Validity and reliability of the parenting stress index of children aged 1 month to 12 years [Thai]. *Journal of Mental Health Thailand*. 28(1), 56–71.

Yodthong, S., Usaha, R., & Poopatayakon, A. (2020). Stress and coping of adolescent mothers in first childbirth [Thai]. *Medical Journal of Sisaket Surin Buriram Hospital.* 35(2), 381–391.

Thailand Ministry of Public Health, Department of Reproductive Health. (2017). *National strategy for the prevention and solution of adolescent pregnancy 2017–2026.* https://rbpho.moph.go.th/upload-file/doc/files/06022019-093301-7565.pdf

Thailand Ministry of Public Health, Department of Reproductive Health. (2022). *Statistics on Adolescent births, Thailand 2022.* https://rh.anamai.moph.go.th/web-upload/7x027006c2abe84e89b5c85b44a692da94/m_magazine/35430/4729/file_download/8ef9ce93951bc46134d960dd8c0106ed.pdf

Abidin, R. R. (1995). *Parenting stress index: Manual (3rd ed.).* Odessa, FL: Psychological Assessment Resources.

Althabe, F., Moore, J. L., Gibbons, L., Berrueta, M., Goudar, S. S., Chomba, E., … McClure, E. M. (2015). Adverse Maternal and Perinatal Outcomes in Adolescent Pregnancies: The Global Network's Maternal Newborn Health Registry Study. Reproductive health, 12(2), 1-9.

Angley, M., Divney, A., Magriple, U., & Kershaw, T. (2015). Social support, family functioning and parenting competence in adolescent parents. *Maternal and Child Health Journal,* 19(1), 67-73.

Barabach, L., Ludington-Hoe, S. M., Dowling, D. & Lotas, M. (2017). Role of baby-friendly hospital care in maternal role competence. *Nursing for Women's Health*, 21(2), 96-107.

Belsky, J. (1984). The determinants of parenting: A process model. *Child Development*, 55(1), 83–96.

Brown, S. G., Hudson, D. B., Grossman, C. C., Kupzyk, K. A., Yates, B. C., & Hanna, K. M. (2016). Social Support, Parenting Competence, and Parenting Satisfaction among Adolescent, African American, Mothers. *Western Journal of Nursing Research*, 1-18.

Corcoran, J. (2016). Teenage Pregnancy and Mental Health. *Societies Journal*, 6, 1-9.

Erfina, E., Widyawati, W., McKenna, L., Reisenhofer, S., & Ismail, D. (2019). Adolescent Mothers' Experiences of the Transition to Motherhood: An Integrative Review. *International Journal of Nursing Sciences*, 6(2), 221-228.

Kershaw, T., Murphy, A., Lewis, J., Divney, A., Albritton, T., Magriples, U., et al. (2014). Family and relationship influences on parenting behaviors of young parents. *Journal of Adolescent Health*, 54, 197-203.

Kroja, R., Latva, R., & Lehtonen, L. (2012). The effects of preterm birth on mother-infant interaction and attachment during the infant's first two years. *Acta Obstet Gynecol Scand*, 91, 164-173.

Maehara, K., Mori, E., Tsuchiya, M., Iwata, H., Sakajo, A., Ozawa, H., et al. (2016). Factors affecting maternal confidence among older and younger Japanese primiparae at one month post-partum. *Japan Journal of Nursing Science*. doi: 10.1111/jjns.12123

Mangeli, M., Rayyani, M., Cheraghi, M. A., & Tirgari, B. (2017). Exploring the Challenges of Adolescent Mothers from Their Life Experiences in the Transition to Motherhood: A Qualitative Study. *Journal of Family and Reproductive Health*, 11(3), 165-173.

Martin, J. A., Hamilton, B. E. & Osterman, M. J. K. (2021). Births in the United States, 2020. *NCHS Data Brief*, 418, 1-8.

Moudi, Z., Talebi, B. & Pour, M. S. (2017). Effect of a brief training program for primigravid adolescents on parenting self-efficacy and mother-infant bonding in the southeast of Iran. *Int J Adolesc Med Health*, 32(1).

Ngai, F. W., & Chan, S. W. C. (2012). Stress, Maternal Role Competence, and Satisfaction among Chinese women in the perinatal period. *Research in Nursing & Health*, 35, 30-39.

Ogawa, K., Matsushima, S., Urayama, K. Y., Kikuchi, N., Nakamura, N., Tanigaki, S., …Morisaki, N. (2019). Association between adolescent pregnancy and adverse birth outcomes, a multicenter cross sectional Japanese study. *Scientific Reports*, 9, 1-8.

Osok, J., Kigamwa, P., Stoep, A. V., Huang, K. Y., & Kuma, M. (2018). Depression and psychosocial risk factors associated with pregnancy in Kenyan adolescents: A cross-sectional study in a community health center of Nairobi. *BMC Psychiatry*, 18(1), 1-10.

Ponomartchouk, D., & Bouchard, G. (2015). New mothers' sense of competence: predictors and outcomes. *Journal of Child and Family Studies*, 24, 1977–1986.

Shrooti, S., Mangala, S., Nirmala, P., Devkumari, S., Dharanidhar, B. (2016). Perceived maternal role competence among the mothers attending immunization clinics of Dharan, Nepal. *International Journal of Community Based Nursing and Midwifery*, 4(2), 100-106.

Shorey, S., Chan, S. W. C., Chong, Y. S., & He, H. G. (2015). Predictors of maternal parental self-efficacy among primiparas in the early postnatal period. *Western Journal of Nursing Research*, 37(12), 1604-1622.

United Nations. (2019). *progress towards the Sustainable Development Goals Report of the Secretary-General*. New York, Economic and Social Council, United Nations.

Vance, A. J., & Brandon, D. H. (2017). Delineating among parenting confidence, parenting self-efficacy, and competence. *Advances in Nursing Science*, 40(4), 18-37.

21. Research Proposal Certification

The researcher hereby certifies to strictly adhere to the procedures and methods outlined in this research proposal.

Sunee.

Signature of Researcher: ....

Affiliation: Department of Maternal, Newborn, and Midwifery Nursing

Institution: Srisavarindhira Thai Red Cross Institute of Nursing

Date: February 1, 2023
